# Radiation-Induced Interfacial Degradation and Mechanical Reliability of Sn-Based Solder Joints: A Systematic Review

**DOI:** 10.3390/ma19194175

**Published:** 2026-09-30

**Authors:** Norliza Ismail, Nurhakimah Norhashim, Wan Yusmawati Wan Yusoff, Sabarina Abdul Hamid, Nadhiya Liyana Mohd Kamal, Zulhilmy Sahwee, Shahrul Ahmad Shah, Atiqah Mohd Afdzaluddin

**Affiliations:** 1 Unmanned Aerial System Research Laboratory, Avionics Section, Malaysian Institute of Aviation Technology, Universiti Kuala Lumpur, Dengkil 43800, Selangor, Malaysia; norliza.ismail@t.unikl.edu.my (N.I.); sabarina@unikl.edu.my (S.A.H.); nadhiyaliyanamk@unikl.edu.my (N.L.M.K.); zulhilmy@unikl.edu.my (Z.S.); shahrul@unikl.edu.my (S.A.S.); 2 Department of Physics, Centre for Defence Foundation Studies, Universiti Pertahanan Nasional Malaysia (UPNM), Kem Sungai Besi, Kuala Lumpur 57000, Malaysia; yusmawati@upnm.edu.my; 3 Institute of Microengineering and Nanoelectronics (IMEN), Universiti Kebangsaan Malaysia, Bangi 43600, Selangor, Malaysia; a.atiqah@ukm.edu.my

**Keywords:** solder joint, radiation effects, microstructural degradation, lead-free alloy, systematic literature review

## Abstract

Solder joints in aerospace, nuclear, and defense electronics are increasingly exposed to ionizing radiation, which creates serious reliability issues. Despite its importance, a comprehensive understanding of the underlying mechanisms and how they vary across radiation types remains lacking. This systematic literature review synthesizes 24 peer-reviewed studies (2000–2025) to evaluate radiation-induced microstructural evolution and mechanical degradation in Sn-based solder alloys. Following PRISMA guidelines, data were analyzed across gamma, electron, proton, neutron, and alpha irradiation, covering Sn–Pb, SAC305, AuSn20, SnBi, and related systems. Gamma irradiation predominantly accelerates interfacial diffusion kinetics, resulting in IMC thickening, oxidation, and shear strength reductions. However, the effects are dose-dependent, where low doses temporarily harden the material, while higher doses make it brittle. Lead-free SAC305 alloys exhibit greater radiation sensitivity than Sn–Pb systems, showing accelerated IMC growth, defect accumulation, and lattice instability. Proton and neutron irradiation induce displacement-dominated damage, including defect clustering and Kirkendall voiding. Combination of electron irradiation and thermal cycling produces synergistic degradation exceeding single-stressor effects. By integrating experimental findings with atomistic and multiscale modeling insights, this review links defect generation, interfacial instability, and mechanical degradation using established radiation-enhanced diffusion (RED) principles. The results clarify radiation-type-dependent damage mechanisms, highlight alloy-specific tolerance differences, and identify critical gaps in multi-stressor testing and predictive lifetime modeling. The mechanistic insights presented here serve as a foundation for optimizing solder alloy design and strengthening qualification procedures for radiation-prone electronic applications.

## 1. Introduction

Solder alloys are critical metallurgical interconnects whose performance depends on microstructure, phase stability, and interfacial reactions. Their reliability is governed by IMC evolution, grain morphology, defect distribution, and diffusion-driven processes within the solder matrix and at the solder–substrate interface [1,2,3,4]. In Sn-based solder systems, particularly SnPb and SnAgCu (SAC) alloys, the formation and growth of IMC such as Cu_6_Sn_5_ and Ag_3_Sn play a significant role in determining mechanical integrity and long-term structural stability [5,6,7,8,9].

Under irradiation environments, solder alloys experience additional thermodynamic and kinetic perturbations arising from energy deposition, atomic displacement, and radiation-induced defect generation. Ionizing radiation including gamma rays, electrons, protons, neutrons, and alpha particles interacts with metallic lattices through mechanisms such as Compton scattering, displacement cascades, and vacancy–interstitial pair formation. These processes introduce nonequilibrium defect populations that enhance diffusion kinetics, alter phase stability, and modify interfacial reaction pathways. Consequently, radiation accelerates IMC growth, oxidation, vacancy clustering, and Kirkendall void formation, degrading solder joint mechanical performance [2,3,4].

The transition from conventional SnPb alloys to lead-free alternatives such as 96.5Sn3.0Ag0.5Cu (SAC305) has further complicated the metallurgical response of solder systems under irradiation [5,6,7,8,9]. Differences in lattice structure, diffusion coefficients, and phase constitution between Pb-containing and Pb-free alloys suggest that radiation tolerance is strongly alloy-dependent. Although many studies report radiation-induced microstructural and mechanical changes, the underlying metallurgical mechanisms remain fragmented [3,5,6,7].

Despite growing experimental evidence, a comprehensive mechanistic interpretation of radiation-induced degradation is still lacking [10,11,12,13,14,15]. A systematic synthesis is therefore required to clarify how radiation type, cumulative dose, and alloy composition collectively dictate microstructural evolution and mechanical degradation in Sn-based solder alloys. This review presents a quantitative synthesis of experimental and modeling studies published between 2000 and 2025, focusing on phase evolution, interfacial kinetics, defect formation, and structure–property relationships in irradiated solder systems. By integrating radiation physics with metallurgical principles, the study establishes a coherent defect-mediated framework for understanding irradiation-driven instability in solder joints.

To address this gap, this systematic literature review (SLR) evaluates radiation type (gamma, proton, neutron, electron, and alpha) effects on the microstructural, mechanical, and electrical behavior of common solder alloys; compares radiation tolerance of lead-based (SnPb), lead-free (SAC305), and specialty systems; and identifies research gaps and future directions for radiation-hardened solder development. Although prior reviews, including our earlier overview of extreme-condition reliability [11], have summarized radiation-induced degradation in solder alloys, most have remained largely descriptive. In contrast, the present work advances the field through a structured, radiation-type-resolved analysis grounded in a defect-driven kinetic perspective. Specifically, it (i) quantitatively combines reported IMC growth and mechanical degradation ranges across different irradiation modes; (ii) systematically contrasts alloy-dependent radiation resistance; and (iii) integrates experimental findings with atomistic and multiscale modeling results to formulate a unified radiation-enhanced diffusion (RED) framework governing microstructural instability. By bringing together radiation physics, defect mechanisms, material kinetics, and reliability, this review offers a complete mechanistic view that has not been compiled before. This framework enables better radiation-aware testing and lays the groundwork for designing more reliable solder alloys for harsh environments.

It is important to distinguish the present work from the authors’ prior publication on radiation effects in solder joints [11]. That earlier paper was a narrative review covering radiation as one of several extreme-condition stressors alongside thermal cycling, vibration, and humidity; it remained largely descriptive and did not employ a structured systematic review methodology. The present work, by contrast, is a Preferred Reporting Items for Systematic Reviews and Meta-Analyses (PRISMA)-compliant systematic literature review focused exclusively on radiation effects across five irradiation modes: gamma, electron, proton, neutron, and alpha. Unlike the prior publication, the current review uses the established RED concept [16,17] to interpret radiation-induced degradation mechanisms in solder joints. In addition, it provides a systematic comparison of different radiation types and alloy systems that was not included in the 2024 narrative review.

## 2. Methodology

This review adopts a SLR methodology in accordance with the PRISMA guidelines to identify, evaluate, and synthesize existing research on the effects of radiation on solder joints. The PRISMA framework ensures transparency, reproducibility, and methodological rigor throughout the review process. The completed PRISMA 2020 checklist, prepared in accordance with the PRISMA 2020 Statement [18], is provided in the Supplementary Materials. The review protocol was registered in the Open Science Framework (OSF) Registry (Registration ID: G26D9).

### 2.1. Research Questions

This systematic literature review was designed to address the following research questions:

- RQ1: What types of radiation have been investigated for their effects on solder joints?
- RQ2: How does radiation influence the microstructural, mechanical, and electrical properties of solder joints?
- RQ3: Which solder alloy compositions demonstrate better resistance to radiation-induced degradation?
- RQ4: What experimental conditions (e.g., radiation dose, energy, and exposure environment) are commonly employed in these studies?
- RQ5: What research gaps remain and require further investigation?

### 2.2. Literature Search Strategy

A comprehensive literature search was conducted using two well-established scientific databases, Web of Science and Scopus, to ensure broad coverage of relevant peer-reviewed studies. The search was limited to journal articles published between 2000 and 2025 and written in English, capturing developments over the past two and a half decades. Because Web of Science and Scopus employ different indexing structures, field tags, and search operators, the Boolean search strings were adapted to match each database’s syntax and retrieval logic while maintaining conceptual consistency (Table 1). Specifically, Web of Science searches were conducted using Title (TI) and Topic (TS) fields with proximity operators (e.g., NEAR/5) to improve precision, whereas Scopus searches were performed using TITLE-ABS-KEY fields without proximity constraints due to database syntax differences. Although the exact Boolean expressions differed slightly between databases, both search strategies were constructed around three core conceptual blocks:

(i) Radiation exposure (e.g., radiation, gamma irradiation, proton irradiation, neutron irradiation),
(ii) Solder materials (e.g., solder joint, SnPb, SAC305, SnAgCu, AuSn, SnBi), and
(iii) Reliability-related properties (e.g., microstructure, mechanical properties, degradation, IMC, failure).

Adjusting the search for each database helped us find relevant studies while avoiding unnecessary results and keeping the method systematic and reliable. Before conducting the full search, both search strings were tested using several well-known studies on radiation effects in solder joints. This step confirmed that the search strings were able to retrieve the main relevant publications from both databases. The initial search yielded 46 records from Web of Science and 52 records from Scopus prior to screening.

### 2.3. Inclusion and Exclusion Criteria

To ensure the selection of only relevant and high-quality studies, clear inclusion and exclusion criteria were established. The inclusion criteria required that studies be peer-reviewed journal articles, published between 2000 and 2025, and written in English. Additionally, the studies needed to focus on the effects of radiation on solder joints, addressing aspects such as microstructural evolution, mechanical properties, electrical characteristics, or overall reliability. Eligible studies also had to employ experimental, simulation, or modeling approaches related to radiation exposure, ensuring that the findings were directly applicable to the research objectives. Conversely, studies were excluded if they were not journal articles, such as conference papers, review articles, theses, or patents. Research that did not directly involve solder joints, including studies that focused solely on semiconductors, integrated circuits, or printed circuit boards without solder, was also omitted. Further exclusion criteria included lack of full-text availability and insufficient technical detail regarding radiation-induced effects. It should be noted that while review articles were excluded as primary data sources to ensure synthesis is based on original experimental and modeling studies, the authors’ prior review [11] informed the conceptual framing and scope of the present work but did not contribute to the primary dataset. By applying these criteria, the final dataset was curated to be both scientifically rigorous and closely aligned with the goals of the study, allowing for a comprehensive and focused analysis of radiation effects on solder joints.

### 2.4. Study Selection Process

The study selection process followed the PRISMA framework and involved multiple screening stages. From the initial 98 records, filtering by publication year, language, and document type reduced the dataset to 66 articles. After removing 8 duplicate records, 58 unique articles remained for title and abstract screening. During this screening stage, articles not directly addressing solder joints or radiation exposure were excluded. As a result, 24 articles were selected for full-text review. Detailed evaluation against the inclusion and exclusion criteria confirmed that all 24 met the required standards and were included in the final synthesis. The complete screening and selection process is summarized in the PRISMA flow diagram (Figure 1), which illustrates the number of records identified, screened, excluded, and included at each stage.

### 2.5. Data Extraction and Synthesis

A structured data extraction process was applied to the final set of 24 selected studies. A standardized extraction table was developed to collect key information, including authorship, publication year, radiation type (e.g., gamma rays, X-rays, neutrons, protons), solder alloy composition (e.g., SnPb, SnAgCu), radiation exposure conditions (dose, energy, duration), and observed effects on solder joint performance. Reported outcomes included changes in microstructure, mechanical strength, electrical resistance, and overall reliability. In addition, the experimental and analytical techniques employed, such as scanning electron microscopy (SEM), energy-dispersive X-ray spectroscopy (EDX), X-ray diffraction (XRD), mechanical testing, and thermal cycling were documented to enable cross-study comparison. The extracted data were synthesized qualitatively to identify recurring trends, variations in experimental methodologies, and correlations between radiation parameters and solder joint degradation. Where appropriate, findings were grouped according to radiation type and solder alloy composition. This structured synthesis provides a coherent overview of the current state of knowledge and highlights key inconsistencies and research gaps, forming the basis for the discussion and conclusions presented in subsequent sections.

## 3. Results

### 3.1. Background of the Selected Articles

Based on the updated screening and inclusion criteria, 24 peer-reviewed experimental studies published between 2000 and 2025 were included in this review. These studies examined the effects of various ionizing radiation sources gamma rays, electrons, protons, neutrons, and alpha particles on different solder joint systems. The temporal distribution of publications and the proportional breakdown by radiation type are shown in Figure 2.

Before interpreting the quantitative trends presented in this review, it is important to acknowledge the substantial heterogeneity of the included studies. The reviewed articles employed different radiation species, energies, dose rates, fluences, exposure durations, solder alloy compositions, and characterization methods. Consequently, direct quantitative comparisons across radiation types should be interpreted with caution. The trends discussed in this review are intended to provide qualitative and semi-quantitative insights rather than statistically representative benchmarks. In addition, a substantial proportion of the reviewed studies originated from two research groups, reflecting the limited size of the current evidence base on radiation effects in solder joints. Therefore, some reported trends require further validation through independent studies and broader research efforts. Throughout this section, qualitative observations such as morphological trends including IMC scallop morphology retention, void formation patterns, and fracture mode transitions are explicitly distinguished from quantitative comparisons, which include reported values such as shear strength reductions (11–37%), hardness measurements (GPa), and dose thresholds.

As illustrated in Figure 2, gamma irradiation represents the major radiation source investigated (75%), followed by electron and proton irradiation, with comparatively limited studies on neutron and alpha exposure. This dominance reflects the practical relevance of Co-60 gamma sources in aerospace, nuclear, and defense-related reliability assessments. In terms of solder material systems, lead-free SAC305 is the most frequently studied alloy, followed by SnPb eutectic solder. Fewer studies address specialty systems such as SnPbSb, AuSn20, SnBi, and SnAg, indicating a research focus on conventional electronic packaging materials. The findings summarized in Table 2 indicate that radiation exposure consistently induces three strongly coupled degradation pathways: microstructural evolution, mechanical degradation, and property instability. Microstructural changes commonly include IMC thickening, radiation-enhanced diffusion, void formation, and oxidation effects. These structural modifications translate into measurable reductions in shear strength and ductility, with alloy-dependent variations in hardness evolution. The quantitative trends extracted from the reviewed studies are synthesized in Figure 3, which compares representative mechanical degradation, IMC growth, and hardness variation across radiation types and solder systems.

As shown in Figure 3a, gamma irradiation generally produces the most pronounced shear strength reduction, particularly in SAC305 and AuSn20 systems. Figure 3b demonstrates substantial IMC thickening under gamma exposure, supporting the role of radiation-enhanced diffusion in accelerating interfacial reactions. Meanwhile, Figure 3c reveals alloy-dependent hardness evolution, where some systems exhibit softening due to defect accumulation, while others show slight hardening effects potentially associated with radiation-induced defect pinning. Collectively, these results confirm that the severity and mechanism of degradation are strongly influenced by radiation type, dose level, and solder composition.

A focused comparison of the gamma irradiation studies was also conducted because gamma irradiation constitutes the largest and relatively most consistent subset of the reviewed literature. Despite differences in dose rate, total dose, and exposure duration, most gamma irradiation studies reported common degradation features, including IMC thickening, oxidation, microvoid formation, and reductions in mechanical performance. SAC305 solder systems generally exhibited more pronounced IMC growth and microstructural evolution, whereas SnPb systems tended to show relatively stable interfacial morphology but remained susceptible to oxidation, microvoid formation, and mechanical degradation under prolonged exposure. These observations provide a more reliable basis for comparison than direct comparisons across different radiation types.

### 3.2. Thematic Analysis of Radiation-Induced Degradation Mechanisms

This section presents a thematic analysis of the reviewed studies to provide a structured discussion of radiation-induced degradation in solder joints. The discussion is organized into five themes: radiation type, solder alloy composition, degradation mechanisms, radiation dose–damage relationships, and the characterization techniques used to evaluate radiation-induced changes.

#### 3.2.1. Influence of Radiation Type

The results summarized in Table 2 demonstrate that gamma radiation is the most extensively studied radiation source affecting solder joint degradation. As shown in Figure 2, gamma irradiation accounts for 16 of the 24 reviewed studies (67%), reflecting both its experimental accessibility and its relevance to aerospace and nuclear environments. Multiple studies on SAC305 and SnPb solder systems consistently report gamma-induced IMC thickening, microvoid formation, oxidation, and mechanical degradation, with shear strength reductions reaching 30–37% under prolonged exposure [8,9]. These findings indicate that gamma irradiation accelerates diffusion-controlled processes at the solder-substrate interface, promoting brittle IMC growth and reducing joint compliance. The degradation mechanisms and exposure conditions differ across radiation types. Radiation affects solder joints through ionization and displacement damage. Gamma rays and electrons primarily interact with matter through ionization processes, generating energetic secondary electrons that create electron-hole pairs and localized defect populations [11]. The resulting defects promote diffusion, accelerating IMC growth and oxidation. Protons and neutrons produce displacement damage, causing defect accumulation, Kirkendall voids, and lattice instability [37].

Comparison between different radiation environments is further complicated by the use of different exposure metrics. Gamma studies typically report dose (Gy), which measures the deposited energy. In contrast, proton and neutron studies usually report fluence (particles cm^−2^), which measures the number of incident particles. In electron irradiation, the absorbed energy may differ from the incident energy due to penetration depth and specimen geometry [38,39]. Therefore, identical nominal irradiation conditions do not necessarily correspond to equivalent defect generation rates or degradation severity. These differences in interaction mechanisms, exposure metrics, and energy deposition behaviour should be considered when comparing degradation trends across different radiation types.

Importantly, several researchers observed non-linear mechanical responses under gamma exposure. Studies on SAC305 by Lehan et al. [27], Ismail et al. [10], and Guan et al. [8] reported an initial increase in hardness or modulus at low radiation doses, followed by a decline at higher doses. This behavior suggests a radiation-induced densification or precipitation hardening effect at early stages, likely driven by enhanced atomic mobility and IMC stabilization. However, with increasing dose or exposure time, the accumulation of defects, microcracks, and oxidation overwhelms these short-term strengthening mechanisms, leading to embrittlement and strength loss. This non-monotonic response highlights a critical limitation of using short-duration irradiation tests for long-term reliability prediction. Compared to gamma radiation, electron irradiation exhibits a more subtle but cumulative degradation mechanism. However, the deposited energy may differ from the incident electron energy depending on sample thickness and electron penetration depth, complicating direct comparison with gamma irradiation studies. Wu et al. [31] and Guan et al. [35] demonstrated that electron exposure alone induces grain coarsening, lattice distortion, and moderate strength degradation. However, when electron irradiation is combined with thermal cycling, degradation accelerates significantly, resulting in substantial shear strength loss and altered fracture modes. This synergy highlights that electron radiation damage is strongly environment-dependent and may be underestimated if evaluated under isothermal conditions only, a critical consideration for spaceborne electronics.

Proton irradiation introduces a distinct degradation pathway dominated by atomic-scale damage rather than visible microstructural failure. Liu et al. [33] reported that proton exposure in SAC305 solder joints increased dislocation density and reduced lattice quality, resulting in a measurable drop in shear strength despite the absence of observable cracks or voids. Comparative studies by Liu et al. [29] further showed that protons induce more severe microstructural and mechanical degradation than electrons at comparable fluence levels, owing to higher momentum transfer and collision cascades. These findings reveal a latent degradation mechanism, where mechanical reliability deteriorates before conventional microstructural indicators become visible.

Although neutron irradiation remains the least investigated radiation type, its impact appears to be potentially severe based on the limited available evidence. Jing et al. [30] demonstrated that neutron exposure generates dense atomic displacement damage and defect clusters, significantly intensifying the Kirkendall effect. Neutron-irradiated solder joints exhibited increased hardness and elastic modulus accompanied by heightened brittleness and premature failure risk, particularly under thermo-mechanical loading. The inclusion of neutron-based studies in this review strengthens the evidence that displacement-dominated radiation sources pose a disproportionate threat to solder joint integrity, even at relatively limited exposure durations.

Based on the literature, radiation type significantly influences both the severity and the underlying mechanisms of solder joint degradation. Gamma radiation primarily accelerates diffusion-driven IMC growth and oxidation; electron irradiation promotes cumulative weakening under cyclic stress; proton irradiation induces hidden lattice-level damage; and neutron irradiation causes severe atomic displacement and brittle failure. Reliance on gamma irradiation alone cannot adequately represent the radiation environments encountered in aerospace, nuclear, and defense applications. Figure 4 schematically summarizes the dominant degradation mechanisms activated by different radiation sources in solder joints, highlighting their distinct microstructural and mechanical impacts.

#### 3.2.2. Effect of Solder Alloy Composition

The evidence in Table 2 shows that solder alloy composition plays a key role in radiation tolerance, often more than radiation dose. SAC305 and SnPb are the most studied solder alloys, allowing a reliable comparison of their radiation tolerance under gamma irradiation, which accounts for 67% of the reviewed studies. Across multiple gamma irradiation studies, SnPb based solders generally exhibited lower degradation levels than lead-free alternatives. However, this finding is based primarily on gamma irradiation studies and may not apply to all irradiation environments. Investigations by Wang et al. [23,24] and Guan et al. [9] showed that eutectic SnPb solder joints retained relatively stable IMC morphology and mechanical performance under prolonged gamma and electron irradiation, with only modest reductions in shear strength. This enhanced stability is widely attributed to the intrinsic ductility of the SnPb matrix, which accommodates radiation-induced defects through plastic deformation rather than crack initiation.

From a mechanistic perspective, the presence of Pb reduces lattice stiffness and suppresses excessive interfacial stress accumulation at the IMC solder boundary. Recent multiscale studies incorporating density DFT and molecular dynamics (MD) simulations further reveal that Pb-rich regions exhibit lower exposure to irradiation-induced amorphization and defect accumulation compared with Sn-rich phases, thereby slowing interfacial degradation under electron and gamma irradiation [34,35]. These atomistic insights provide a mechanistic explanation for the experimentally observed crack resistance and delayed brittle failure in SnPb solder joints, reinforcing their suitability for radiation-intensive environments where regulatory constraints permit their use. In contrast, lead-free SAC305 solder joints were consistently found to be more vulnerable to radiation-induced degradation, particularly under gamma and proton exposure. Multiple studies reported pronounced IMC overgrowth involving Ag_3_Sn and Cu_6_Sn_5_, eutectic phase redistribution, oxidation, and microcrack formation near the IMC–solder interface [6,8,10,27]. Some nanoindentation studies found increased hardness at low radiation doses, but this reflects local micromechanical behaviour. A reduction in hardness was observed at higher doses and was associated with defect accumulation, void formation, and microstructural instability [5,28]. The radiation response therefore depends on both the property being measured and the irradiation conditions.

Proton irradiation studies by Liu et al. [29,33] further underscored the weakness of SAC305 alloys. Proton-irradiated SAC305 solder joints exhibited measurable reductions in shear strength (~9–10%) and degraded lattice quality, even in the absence of visible microcracks or voids. These findings indicate that mechanical weakening can occur before observable microstructural failure. This latent damage mechanism poses a significant reliability risk for long-duration space missions, where cumulative proton fluence is unavoidable and inspection opportunities are limited.

A comparative summary of alloy performance across multiple degradation criteria is drawn from the data in Table 2. Based on the reviewed studies, SAC305 demonstrates good-to-excellent resistance in most categories but exhibits vulnerability in oxidation resistance, consistent with the accelerated oxidation reported by Guan et al. [8]. In contrast, SnPb solders demonstrate superior performance across nearly all assessed criteria under gamma irradiation, reinforcing their relative radiation tolerance compared with lead-free alloys under this radiation type [9,23]. It should be noted that insufficient data exist for proton, neutron, or electron irradiation of both alloy systems under comparable conditions to extend this conclusion beyond gamma exposure.

Specialty and high-performance solder alloys, including AuSn20 and SnBi, also exhibit pronounced radiation sensitivity. Wen et al. [22] reported severe void growth, excessive IMC thickening, and a transition from ductile to brittle fracture in AuSn20 solder joints, resulting in shear strength reductions exceeding 37% after prolonged gamma irradiation. Notably, combined gamma irradiation and thermal cycling further accelerated degradation in AuSn20 systems, indicating limited tolerance to coupled environmental stresses [12]. Recent studies on SnBi eutectic solder joints revealed radiation-induced oxidation corrosion driven by vacancy formation and enhanced oxygen adsorption, leading to continuous SnO_2_ layer thickening and surface embrittlement under gamma exposure [36]. These findings demonstrate that high-melting-point or noble-metal-containing solders are not inherently radiation-resistant and may experience accelerated interfacial degradation due to complex oxidation and diffusion pathways.

The literature demonstrates that SnPb alloys exhibit the highest radiation tolerance, followed by SAC305 with pronounced dose-dependent instability, while specialty alloys such as AuSn20 and SnBi show severe brittleness and oxidation-driven degradation. The currently available literature suggests that SnPb alloys may exhibit comparatively lower degradation than SAC305 under gamma irradiation conditions. However, additional evidence from proton, neutron, electron, and mixed-radiation environments is required before establishing definitive alloy rankings. Nevertheless, the reviewed studies clearly demonstrate that alloy composition strongly influences radiation-induced degradation behaviour. This observation highlights the need for radiation-aware solder alloy design, including microalloying strategies, IMC growth suppression, and composite or functionally graded solder systems, to reconcile environmental compliance with the reliability requirements of aerospace, nuclear, and defense electronics. Table 3 summarises the dominant degradation mechanisms, representative property changes, and dose ranges across radiation types and alloy systems.

#### 3.2.3. Degradation Mechanisms

According to the findings summarized in Table 2, radiation-induced degradation of solder joints can be systematically classified into three strongly coupled mechanisms: microstructural evolution, mechanical degradation, and electrical–thermal instability. These mechanisms are causally linked: radiation-induced defects initiate microstructural changes that progressively translate into mechanical weakening and functional failure.

Microstructural evolution represents the primary and initiating degradation pathway under radiation exposure. Across gamma-irradiated SAC305 and SnPb systems, numerous studies reported accelerated IMC growth, eutectic phase redistribution, oxidation, and void formation at the solder–substrate interface [8,10,27]. These effects are widely attributed to radiation-enhanced diffusion, where point defects such as vacancies act as fast diffusion pathways, promoting interfacial reactions even at relatively low temperatures. Prolonged gamma exposure further induces surface and interfacial oxidation, including the formation of SnO_2_ nanocrystals, which destabilize the solder matrix and facilitate crack initiation [9,32].

In gamma-irradiated SAC305 and SnPb solder systems, the interfacial IMC layer is predominantly composed of Cu_6_Sn_5_ and generally retains its characteristic scallop-like morphology while undergoing progressive thickening with increasing radiation dose and exposure duration [7,21,28]. In several studies, IMC growth was accompanied by interfacial roughening and localized void formation, indicating accelerated diffusion processes at the solder–substrate interface.

Although both Guan et al. [8] and Lehan et al. [27] investigated SAC305 solder systems under gamma irradiation, oxidation was only explicitly reported by Guan et al. [8]. This discrepancy may be attributed to differences in irradiation conditions, environmental factors, specimen preparation, and characterization sensitivity. Guan et al. [8] employed advanced TEM and surface-sensitive analyses capable of detecting oxidation products, whereas oxidation was not reported by Lehan et al. [27]. It is therefore possible that differences in analytical sensitivity and experimental conditions contributed to the contrasting observations. These findings highlight the importance of standardized characterization protocols when evaluating radiation-induced oxidation behaviour in solder joints.

By comparison, proton and neutron irradiation studies have mainly focused on defect accumulation and lattice damage, with limited information on IMC morphology and thickness [30,33]. Proton irradiation generates high dislocation densities and degrades the lattice quality of SAC305 solder joints without immediately producing visible cracks or voids [33]. Neutron irradiation, although less frequently studied, produces dense defect clusters and vacancy accumulation, intensifying the Kirkendall effect and promoting interfacial instability [30]. These findings suggest that different radiation types produce different degradation mechanisms and IMC evolution.

Mechanical degradation emerges as a direct consequence of accumulated microstructural damage. Nearly all studies in Table 2 report reductions in shear strength, tensile strength, hardness, or ductility following radiation exposure. For example, AuSn20 solder joints exhibited shear strength reductions exceeding 37% after prolonged gamma irradiation, accompanied by a transition from ductile to brittle fracture behavior [22]. Similar strength losses were observed in SAC305 joints subjected to combined electron irradiation and thermal cycling, highlighting the importance of synergistic stress effects [31].

Several investigations also reported non-linear mechanical responses, where hardness or strength initially increased at low radiation doses before declining at higher doses [5,10,28]. This transient strengthening has been attributed to defect pinning and IMC densification; however, as radiation exposure continues, defect clustering, void coalescence, and interfacial cracking dominate, leading to rapid embrittlement. Atomistic and multiscale modeling studies further reveal that radiation-induced defects weaken Sn-rich phases and alter interfacial bonding, accelerating crack initiation along the IMC–solder interface [34,35]. This explains why excessive IMC growth often results in uneven mechanical degradation relative to bulk microstructural changes.

Although less addressed, electrical and thermal instability are also caused by long-term degradation. Wu et al. [31] provided preliminary evidence that radiation-induced microstructural damage disrupts conductive pathways and heat dissipation within solder joints, reporting increased electrical resistance and elevated resistor temperatures in electron-irradiated SAC305 joints, particularly under combined thermal cycling. These changes are linked to void formation, oxidation, and microcracking, which locally increase resistivity and promote thermal accumulation [40,41]. While the current evidence base is limited to this single study, the findings indicate that electrical reliability deserves greater attention in future radiation-effects research, particularly for power electronics and high-frequency systems where even minor resistance increases can compromise performance.

#### 3.2.4. Correlation Between Radiation Dose and Damage

The findings summarized in Table 2 demonstrate that radiation-induced damage in solder joints is strongly dose dependent but inherently non-linear, reflecting the competing effects of defect generation, defect interaction, and microstructural evolution. Rather than exhibiting monotonic degradation with increasing exposure, many solder systems display a threshold-type response, in which moderate radiation doses induce transient strengthening before progressive deterioration dominates at higher cumulative doses or longer exposure durations. This non-linear dose–response relationship is illustrated schematically in Figure 5, which captures the characteristic transition from initial hardening to eventual embrittlement.

Multiple gamma irradiation studies on SAC305 and SnPb solder joints reported initial increases in hardness or shear strength at low to intermediate doses, typically within the range of ≤15 Gy [5,10,28]. This behavior has been attributed to radiation-enhanced diffusion and defect pinning, which temporarily stabilize IMC layers and restrict dislocation motion. Similar early-stage strengthening has also been observed under electron irradiation in SnPb systems, where irradiation-induced electron localization effects delay plastic deformation [34,35]. However, these strengthening effects are transient and should not be interpreted as improved long-term reliability. This complexity is reflected in the alloy-level trends summarized in Table 2, which capture overall radiation tolerance rather than transient responses.

As radiation exposure increases beyond a critical threshold, damage accumulation consistently outweighs microstructural stabilization. High-dose gamma irradiation promotes excessive IMC thickening, oxidation, void nucleation, and interfacial cracking, leading to substantial mechanical degradation. Reported shear strength reductions range from approximately 11% to 37% across all studies synthesized in Table 2, depending on alloy composition and irradiation duration, with the most severe degradation (>30%) observed in AuSn20 joints under prolonged gamma exposure [22,27]. This transition from apparent hardening to rapid degradation reflects the evolution of isolated point defects into defect clusters, microvoids, and crack initiation sites that irreversibly weaken the solder joint.

Fluence-controlled studies under electron and proton irradiation further emphasize the importance of cumulative exposure history. Proton irradiation in SAC305 solder joints resulted in measurable reductions in shear strength (≈9–10%) and degraded lattice quality even in the absence of visible microcracks or voids [33]. Comparative studies demonstrated that protons induce more severe degradation than electrons at comparable fluence levels due to higher momentum transfer and enhanced atomic displacement damage [29]. These results indicate that dose rate alone is an insufficient metric for reliability assessment, particularly for long-duration space applications characterized by sustained low-dose irradiation. The displacement-dominated damage from proton exposure is not captured by gamma-only testing protocols, underscoring the need for radiation-type-specific qualification. Alpha irradiation, while the least studied radiation type in the reviewed literature, is represented by a single study on low-alpha SAC105 solder [19]. That study demonstrated that reducing alpha particle emission refines solder microstructure and improves wetting behavior, with the primary reliability concern being soft error induction in adjacent semiconductor devices rather than structural solder joint degradation. Although definitive conclusions on alpha-induced mechanical or microstructural degradation cannot be drawn from this limited evidence base, the distinct emission-reduction mechanism warrants dedicated experimental investigation, particularly for memory-intensive applications operating in alpha-rich environments.

Quantitative analysis of the reviewed studies reveals distinct dose thresholds across radiation types and alloy systems. For gamma-irradiated SAC305, transient hardening typically peaks at 10–15 Gy, with mechanical degradation accelerating above 50 Gy cumulative dose [5,10,27]. SnPb systems exhibit broader tolerance, with significant strength reductions generally requiring exposure >500 Gy or prolonged irradiation >500 h [7,9]. Electron irradiation effects become pronounced at fluences exceeding 10^16^ e/cm^2^ [34], while proton-induced damage is detectable at fluences as low as 10^11^ p/cm^2^ [33]. These thresholds, summarized in Table 3, provide preliminary benchmarks for radiation qualification testing.

The non-linear dose–damage relationship observed across different radiation types arises from the dynamic evolution of radiation-induced defects. At low doses, isolated vacancies and dislocations may recombine or contribute to short-term IMC stabilization. At higher cumulative doses, however, defect clustering, void coalescence, and dislocation pile-up dominate, leading to brittle fracture behavior and interfacial failure. Neutron irradiation exacerbates this process by producing dense displacement cascades, resulting in mechanically stiff yet brittle solder joints with elevated failure risk under thermomechanical loading [30].

Overall, these findings confirm that radiation dose cannot be treated as a simple linear predictor of solder joint reliability. Instead, dose rate, cumulative fluence, exposure duration, and radiation type must be jointly considered, along with synergistic environmental stressors such as thermal cycling. Figure 5 summarizes the characteristic dose–response trajectory observed across multiple studies, while Table 3 provides quantitative comparisons across radiation types. Failure to account for these factors may lead to significant overestimation of solder joint durability in radiation-rich environments. This dose-dependent behavior, particularly the latent damage mechanisms revealed by proton irradiation, informs the mechanistic framework discussed in Section 4.

#### 3.2.5. Characterization Techniques

As summarized in Table 2, studies investigating radiation effects on solder joints employ a combination of microstructural, mechanical, and functional characterization techniques, with a predominant focus on post-irradiation microstructural analysis. The techniques discussed below provide experimental evidence supporting the findings presented throughout this review, including the dose-dependent trends discussed in Section 3.2.4.

Among these techniques, SEM is the most widely used across different radiation types and solder alloy systems. It enables direct visualization of IMC morphology, layer thickness, void formation, and crack initiation at the solder–substrate interface [42,43,44,45]. Studies on SAC305, SnPb, and AuSn20 consistently show radiation-induced IMC growth and crack propagation, linking microstructural evolution to mechanical degradation [8,10,12,22]. Representative SEM images are presented in Figure 6, illustrating IMC thickening, void formation, crack propagation, and surface oxidation.

To complement morphological observations, energy-dispersive X-ray spectroscopy (EDS/EDX) is frequently employed to examine elemental redistribution and interfacial reactions [46]. EDS analysis has been particularly effective in identifying radiation-enhanced diffusion of Cu, Ag, Au, and Ni into the solder matrix, as well as tin depletion and oxygen enrichment associated with irradiation-induced oxidation [8,9]. In SnPb and SnBi systems, EDS provided critical evidence of Pb segregation, Sn oxidation, and Bi-assisted oxidation pathways under gamma irradiation, supporting mechanistic interpretations that cannot be inferred from morphology alone [32,36].

X-ray diffraction (XRD) is selectively used to investigate phase stability, lattice distortion, and crystallographic changes induced by radiation exposure [47,48,49,50]. Proton and gamma-irradiated solder joints commonly exhibit peak broadening, intensity variation, and the emergence of oxide-related phases, indicating increased lattice strain and defect density [10]. Although XRD helps detect crystal structure damage caused by radiation, it cannot easily identify very small, localized damage at interfaces due to its lower spatial resolution. This is why it is used less often than SEM-based techniques. However, XRD results showing lattice distortion still indirectly support the displacement damage mechanisms discussed in Section 3.2.1.

Mechanical characterization is a main component of radiation reliability assessment. Shear testing is the most reported mechanical evaluation method, serving as a direct indicator of joint integrity and load-bearing capability. Across multiple studies, shear strength reductions ranging from approximately 11% to 37% were observed following radiation exposure, depending on alloy composition, radiation type, and dose as synthesized in Section 3.2.4 and Table 3 [7,12,31]. Nanoindentation and microhardness testing are increasingly adopted to probe localized mechanical responses within solder matrices and IMC regions [51,52,53]. These techniques revealed non-linear hardness evolution, including transient hardening at low radiation doses followed by softening at higher doses. This behavior reflects the opposing effects of defect pinning and microcrack formation due to irradiation, as illustrated in Figure 6.

Despite their importance, electrical and thermal characterization techniques remain underexplored in the literature. Only a limited number of studies incorporated electrical resistance measurements or thermal monitoring to evaluate functional degradation following irradiation [31]. Available evidence indicates that radiation-induced voids, oxidation, and microcracks disrupt conductive pathways and impair heat dissipation, leading to increased resistance and localized temperature rise [54,55,56]. Electrical and thermal reliability remain among the least investigated aspects of radiation effects on solder joints. This lack of data highlights a significant research gap, particularly because solder joints serve both mechanical and electrical functions.

Several studies used combined environmental testing, such as radiation exposure followed by thermal cycling, to better simulate real service conditions. These studies demonstrated accelerated degradation compared with radiation-only tests, highlighting the importance of considering combined stress effects. However, differences in testing methods remain a major limitation. Studies vary widely in irradiation conditions, sample geometry, joint size, and reporting methods. In addition, most research relies on post-irradiation analysis, which only provides static observations of damage and does not capture real-time defect formation or crack growth. These gaps, summarized in Figure 7, limit comparison between studies and highlight the need for standardized testing protocols. Future studies should incorporate advanced characterization techniques such as in situ TEM, synchrotron X-ray diffraction, atom probe tomography (APT), and three-dimensional X-ray tomography. These methods provide direct insight into defect evolution, diffusion behaviour, and interfacial degradation. Unlike conventional post-irradiation analysis, such approaches enable real-time observation of damage evolution and crack initiation.

Current techniques can detect radiation-induced microstructural and mechanical degradation, but the lack of standardized protocols, limited electrical assessment, and absence of in situ methods reduce comparability and predictive accuracy. The techniques reviewed here have provided the empirical foundation for the dose thresholds in Table 3. Future work should develop standardized, multi-parameter frameworks integrating microstructural, mechanical, electrical, and thermal evaluations, as highlighted in Section 3.2.6.

#### 3.2.6. Research Gaps and Limitations

Although many studies have examined radiation effects on solder joints [1,11], several important research gaps and methodological limitations remain, as summarized in Table 2. Figure 7 provides a visual summary of these gaps, organized by category, which are elaborated below. As a result, current findings may not fully reflect real-world radiation conditions.

A major imbalance exists in the coverage of radiation types. Most studies focus on gamma irradiation while electron, proton, and neutron radiation remain significantly underexplored, despite their relevance to space, high-altitude, and nuclear environments [29,30,31,32,33,57,58]. Neutron irradiation studies are limited, even though neutrons are known to induce severe atomic displacement damage and defect clustering. As quantified in Table 3, proton and neutron irradiation produce displacement-dominated damage mechanisms that are fundamentally distinct from the diffusion-driven effects of gamma exposure. This limited coverage constrains understanding of displacement-dominated degradation mechanisms and introduces uncertainty in reliability predictions for environments where neutron or proton flux is significant. Alpha irradiation represents the most critical coverage gap: only one study in the reviewed literature addresses alpha effects on solder joints (Study 1, SAC105) [19] and no data exist on alpha-induced structural damage mechanisms in SnPb or SAC305 systems. Given that alpha-emitting isotopes are present in certain nuclear and space environments [59] dedicated experimental investigation of alpha-induced microstructural degradation is strongly recommended as a priority for future work. Future studies should adopt standardized reporting of irradiation parameters, including dose, dose rate, fluence, energy level, exposure duration, and specimen geometry, to enable meaningful comparison across different radiation types and experimental conditions.

Another major limitation is the reliance on single-stressor experiments. In practice, electronic assemblies are exposed to combined radiation, thermal, mechanical, and environmental stresses. Only a small number of studies in Table 2 have investigated the combined effects of radiation and thermal cycling. These studies consistently reported greater degradation than single-stressor tests [12,31]. Wu et al. [31] observed a 28.0% reduction in shear strength under combined electron irradiation and thermal cycling compared with individual stressors. This highlights the limitations of single-stressor testing in predicting long-term damage accumulation. Beyond combined thermal-radiation testing, future studies should also investigate co-irradiation environments involving simultaneous exposure to gamma rays and particle radiation (protons, neutrons, or electrons).

Unlike single-radiation experiments, co-irradiation may generate complex defect interactions through the combined action of ionization-induced and displacement-induced damage mechanisms [60,61]. Such defect mixing could modify vacancy-interstitial recombination, diffusion kinetics, oxidation behaviour, IMC growth, and mechanical degradation pathways. Since practical aerospace, defence, and nuclear environments often involve multiple radiation species simultaneously, co-irradiation testing represents a critical research priority for future qualification frameworks. Establishing the relative contribution of ionization and displacement damage under co-irradiation conditions would significantly improve the predictive capability of radiation reliability models for advanced electronic packaging systems.

From a materials perspective, existing research remains heavily concentrated on SnPb and SAC305 solder systems, with limited exploration of alternative lead-free alloys, low-temperature solders, or emerging interconnect materials used in advanced packaging technologies [19,26,62]. In contrast, specialty alloys such as AuSn20 and SnBi are less frequently studied, and their consistently limited performance documented in the reviewed studies is based on very few data points. In addition, fine-pitch interconnects, micro-bumps, and heterogeneous integration platforms are rarely included in radiation reliability studies. Because of this limited focus on materials and architectures, current conclusions may not fully apply to next-generation electronic systems.

Significant methodological inconsistencies further complicate cross-study comparison. Radiation dose, fluence, dose rate, exposure duration, irradiation atmosphere, and specimen geometry vary widely across studies, and in many cases are insufficiently reported [8,30,33,58]. Table 3 highlights this variability: gamma doses span five orders of magnitude (5 Gy to 800 kGy), and neutron fluence is not explicitly reported in the sole available study. The absence of standardized irradiation and testing protocols hampers reproducibility and prevents meaningful quantitative benchmarking. In addition, most studies rely exclusively on post-irradiation characterization, providing only static snapshots of damage evolution and obscuring the kinetics of defect formation, crack initiation, and phase transformation.

Another important limitation is the near absence of in situ characterization during irradiation. Most studies rely exclusively on post-irradiation observations, preventing direct monitoring of defect generation, IMC evolution, oxidation kinetics, and crack initiation. Advanced in situ techniques such as synchrotron X-ray diffraction, environmental TEM, real-time SEM, and electrical resistance monitoring could provide critical mechanistic insights that are currently unavailable. A further limitation is the limited availability of electrical and thermal reliability data [31,63,64]. While mechanical degradation is extensively documented, relatively few studies evaluate changes in electrical resistance, thermal conductivity, or signal integrity following irradiation. This gap is particularly important because solder joints function not only as mechanical interconnects but also as main electrical pathways within electronic systems.

Finally, although recent studies have begun to incorporate atomistic and multiscale simulations, mechanism-based predictive modeling remains limited [34,35,36,58]. Few works integrate experimental data with validated models capable of forecasting long-term degradation or failure under cumulative radiation exposure. The dose thresholds listed in Table 3 provide useful experimental data, but they have not yet been used in predictive lifetime models. Without these predictive frameworks, it is difficult to develop reliable lifetime assessment tools and radiation-aware design guidelines. All these considerations should be incorporated into future experimental designs and reliability qualification frameworks to ensure that testing conditions more accurately represent operational environments and provide more reliable lifetime assessment data.

## 4. Discussion

This review synthesizes experimental and modeling evidence from 24 studies (summarized in Table 2) to elucidate how radiation type, cumulative exposure, and solder alloy composition govern solder joint reliability in radiation-intensive environments [1,11,58]. Rather than repeating individual degradation observations, the following discussion integrates the key findings presented in Section 3, including radiation-type effects, alloy-dependent responses, degradation pathways, dose–damage relationships, and the identified research gaps, to highlight their implications for materials selection, reliability assessment, and qualification strategies.

### 4.1. Radiation-Type Dependence of Degradation Mechanisms

Degradation mechanisms are fundamentally radiation-type specific. Gamma irradiation predominantly accelerates diffusion-driven processes, including intermetallic compound growth, oxidation, and void formation, leading to progressive interfacial embrittlement (as illustrated in Figure 4 and quantified by the IMC growth rates in Table 3) [8,9,57]. In contrast, electron and proton irradiation introduce displacement-driven lattice damage that may not immediately manifest as visible microstructural failure yet measurably degrades mechanical integrity a latent damage mechanism documented in Section 3.2.1 and reflected in the shear strength reductions compiled in Table 3 [29,33]. Neutron irradiation, though less frequently investigated, produces the most severe displacement damage, promoting defect clustering and brittle behavior [30]. These distinctions demonstrate that reliance on gamma irradiation alone is insufficient to represent the complex radiation environments encountered in aerospace, nuclear, and defense electronics [49]. Table 3 provides a quantitative basis for selecting qualification protocols matched to mission-specific radiation spectra.

### 4.2. Alloy-Dependent Radiation Tolerance

Alloy composition is a dominant determinant of radiation tolerance. Sn–Pb solder joints consistently exhibit greater resilience under irradiation due to their inherent ductility and reduced susceptibility to irradiation-induced amorphization a finding consistent with the quantitative data consolidated in Table 2 [23,24]. Lead-free SAC305 systems, while environmentally compliant, display pronounced non-linear degradation behavior characterized by transient hardening at low doses followed by accelerated embrittlement at higher cumulative exposures (see dose–response trajectory in Figure 5) [5,10,27,28]. Specialty alloys such as AuSn20 and SnBi exhibit limited tolerance to radiation-induced oxidation and interfacial instability, as documented in the reviewed studies and the oxidation mechanisms elucidated in Section 3.2.3. These findings underscore a trade-off between environmental regulation and long-term reliability that must be addressed through radiation-aware alloy design. The limited dataset for specialty alloys (Section 3.2.6, Figure 7) highlights the urgent need for additional testing.

### 4.3. Non-Linear Dose Effects and Qualification Implications

The non-linear relationship between radiation dose and damage characterized by transient hardening followed by embrittlement represents a main challenge for reliability qualification. Short-term strengthening effects observed at low doses (typically ≤15 Gy for SAC305, as documented in Section 3.2.4) may mask underlying defect accumulation, leading to overestimation of solder joint durability if cumulative fluence and exposure duration are not considered [5,10,27,28]. Proton and electron irradiation studies further reveal that latent lattice-level damage can significantly reduce strength prior to the appearance of microstructural indicators a phenomenon captured in the dose thresholds of Table 3. This behavior highlights the inadequacy of single-dose or short-duration testing for predicting long-term service performance [16,57]. The dose–response data synthesized in Table 3 provide preliminary thresholds for designing qualification tests that capture both transient hardening and degradation phases.

### 4.4. The Radiation-Enhanced Diffusion (Red) Framework

The degradation phenomena observed across different radiation types can be fundamentally interpreted through the lens of radiation-induced defect generation and RED. This framework provides a unified mechanistic basis for understanding the diverse observations reported in Section 3 [16,34,35]. When energetic photons or particles interact with metallic lattices, energy deposition leads to the formation of nonequilibrium point defects, primarily vacancies and self-interstitial atoms [16]. In displacement-dominated environments such as proton and neutron irradiation, collision cascades generate defect clusters and localized lattice disorder [30,33,57]. Even in gamma irradiation, secondary electron production through the Compton effect introduces sufficient defect populations to perturb diffusion-controlled processes [8,23].

From a metallurgical standpoint, the introduction of excess vacancies significantly alters atomic mobility within Sn-based solder matrices. Vacancy supersaturation reduces effective diffusion activation energy, thereby accelerating interfacial reactions between solder and substrate. This mechanism explains the frequently reported thickening of Cu_6_Sn_5_ and Ag_3_Sn IMCs under irradiation [8,10,17]. Unlike thermally driven diffusion, radiation-enhanced diffusion occurs under relatively low macroscopic temperature conditions, indicating that defect flux rather than thermal energy becomes the dominant kinetic driver [16,17]. Although vacancy-mediated diffusion is widely considered the dominant contributor to RED, interstitial defects may also influence radiation-induced microstructural evolution. Interstitial clustering can modify defect recombination kinetics, local stress fields, and atomic transport pathways, potentially affecting phase stability and interfacial degradation. However, direct experimental evidence regarding interstitial behaviour in irradiated solder systems remains limited. Further investigation is therefore required to clarify the role of interstitial defects in radiation-induced degradation mechanisms.

In multiphase solder systems, radiation-induced diffusion asymmetry further intensifies the Kirkendall effect. Enhanced outward diffusion of Sn relative to Cu or Ag promotes vacancy accumulation at the interface, leading to microvoid nucleation and eventual coalescence. Over time, these voids evolve into crack initiation sites, particularly along brittle IMC layers [65]. This interfacial instability is especially pronounced in SAC305 alloys, where higher IMC volume fraction and stiffer intermetallic phases increase stress concentration under mechanical loading [5,9]. The alloy-dependent radiation tolerance observed between SnPb and lead-free SAC systems can also be rationalized metallurgically. The presence of Pb introduces a softer matrix phase with higher defect recombination capability and lower lattice stiffness, enabling more effective accommodation of irradiation-induced strain. In contrast, Sn-rich lead-free alloys exhibit stronger directional bonding and higher elastic modulus, which may limit defect annihilation and promote defect clustering [23,34]. Atomistic simulations reported in recent studies further indicate that Sn-rich phases possess lower resistance to irradiation-induced amorphization compared to Pb-rich regions [32,35], supporting experimental observations of earlier embrittlement in SAC alloys [5,8,10].

Oxidation phenomena observed under prolonged gamma irradiation can likewise be interpreted through defect-mediated mechanisms. Radiation-induced vacancies enhance oxygen adsorption and diffusion, facilitating SnO_2_ nucleation at free surfaces and interfaces [8,9]. Once formed, oxide layers alter interfacial bonding and modify stress distribution within the solder joint. The coupling between oxidation kinetics and IMC evolution therefore represents a synergistic degradation pathway rather than an isolated surface reaction [35,36]. This mechanism is consistent with the vacancy-enhanced oxidation reported for SnBi solder joints [36] and discussed in Section 3.2.3.

These findings indicate that radiation-induced degradation in solder alloys is governed by a defect-driven kinetic regime in which vacancy generation, diffusion acceleration, interfacial reaction kinetics, and phase stability become strongly coupled [29,35,36]. The transition from transient hardening at low doses to pronounced embrittlement at higher cumulative exposure captured in Figure 5 can be interpreted as the evolution from isolated point defects to defect clustering, void formation, and interfacial decohesion [5,10,28]. This metallurgical framework provides a mechanistic foundation for designing radiation-tolerant solder alloys through microstructural control, defect recombination enhancement, and IMC growth suppression strategies [58,65]. This RED-based interpretation helps explain the observations summarized in Table 3 and provides useful insight into alloy-dependent radiation responses and future radiation-aware solder development [11].

### 4.5. Synthesis and Path Forward

Table 3 consolidates the radiation-type-specific damage pathways and alloy-dependent reliability trends identified throughout this review, providing a quantitative reference for designing qualification tests and interpreting experimental results. The collective evidence establishes that radiation-induced degradation in solder joints cannot be characterised by a single mechanism, dose metric, or alloy response. Gamma, electron, proton, and neutron irradiation each activate distinct kinetic pathways such as diffusion-driven, displacement-dominated, or oxidation-mediated, that interact differently with alloy microstructure and evolve non-linearly with cumulative dose. Reliable qualification therefore demands radiation-type-specific testing protocols, alloy selection informed by mechanistic tolerance criteria, and multi-stressor experimental frameworks that reflect operational reality rather than isolated laboratory conditions.

Future research should focus on the key gaps identified in Section 3.2.6 (Figure 7). These include broader radiation coverage, standardized testing, diverse solder systems, advanced characterization, and predictive reliability modeling. These priorities can be guided by the RED framework elaborated in Section 4.4. By attributing the full spectrum of observed degradation, including IMC overgrowth, Kirkendall voiding, oxidation, and non-linear dose response, to a common defect-driven kinetic origin, RED provides a physically grounded basis for radiation-aware alloy design, microstructural optimisation, and qualification standard development in aerospace, nuclear, and defence electronics.

## 5. Conclusions

This systematic review of 24 experimental studies (2000–2025) establishes how radiation type, cumulative dose, and alloy composition collectively influence solder joint degradation. Radiation type dictates both the dominant degradation mechanism and its severity. Gamma irradiation primarily accelerates diffusion-driven processes such as IMC growth, oxidation, and eutectic instability, whereas proton, electron, and neutron irradiation introduce varying degrees of defect accumulation and lattice disorder. The limited available evidence suggests that neutron irradiation may produce severe displacement-driven degradation, although further studies are required to validate this observation. Alloy composition strongly influences radiation response. Existing gamma irradiation studies suggest that SnPb solder systems may exhibit lower degradation than SAC305 under comparable exposure conditions. However, insufficient evidence currently exists to establish a universal ranking of radiation tolerance across different irradiation environments. SAC305 alloys generally exhibit dose-dependent behaviour characterized by microstructural instability, oxidation, and progressive mechanical degradation at higher radiation exposures. The reviewed studies collectively support the application of established RED principles to interpret radiation-induced degradation in solder joints. Nevertheless, important research gaps remain, including the limited availability of neutron, proton, electron, alpha-particle, and mixed-radiation studies, the lack of in situ characterization techniques, and the scarcity of electrical and thermal reliability assessments. Addressing these gaps will support the development of radiation-aware solder alloys and more reliable qualification strategies for electronic systems operating in harsh radiation environments.

## Figures and Tables

**Figure 1:**
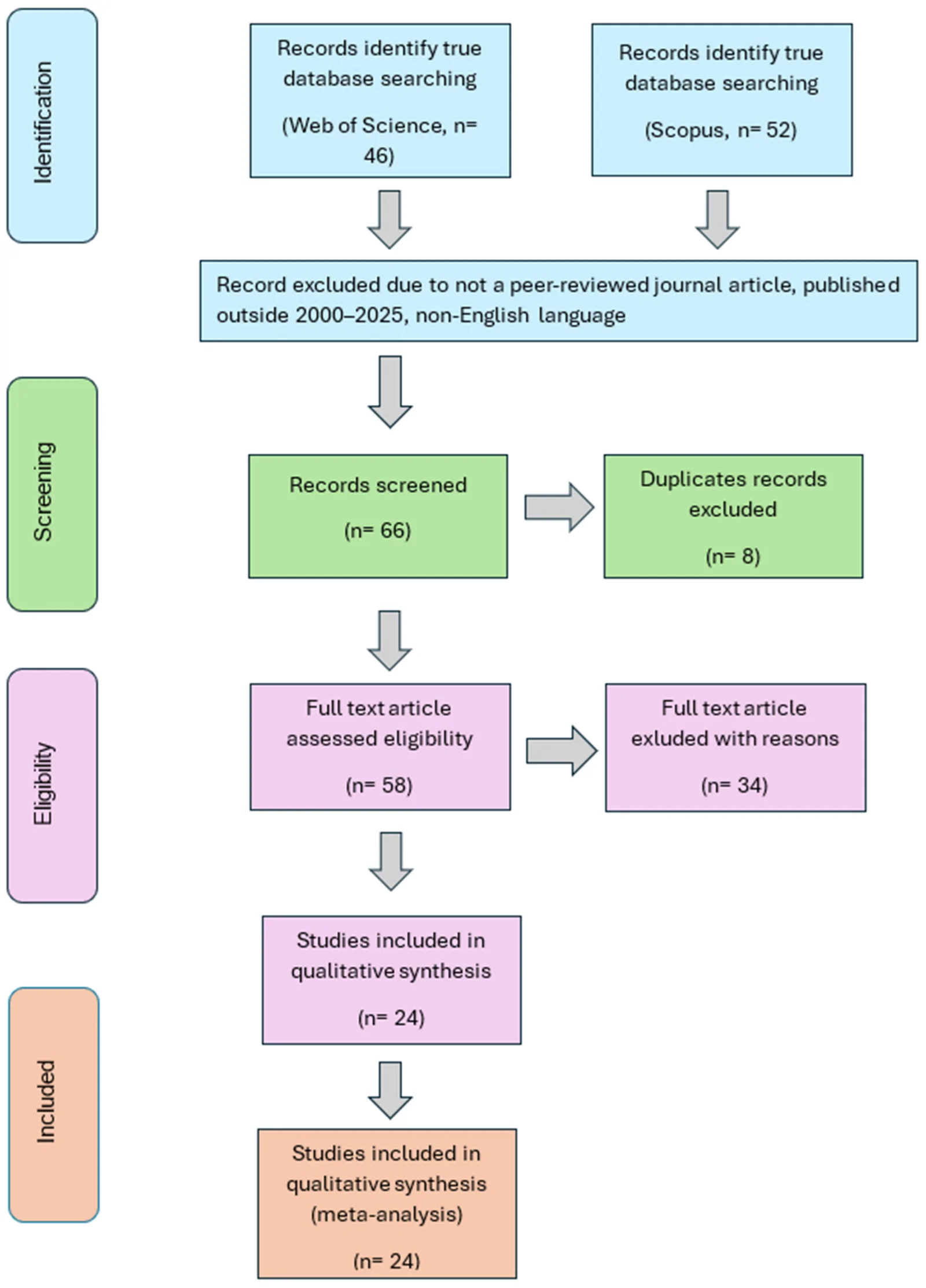
PRISMA flow diagram illustrating the study selection process for the systematic review of radiation effects on solder joints (2000–2025).

**Figure 2:**
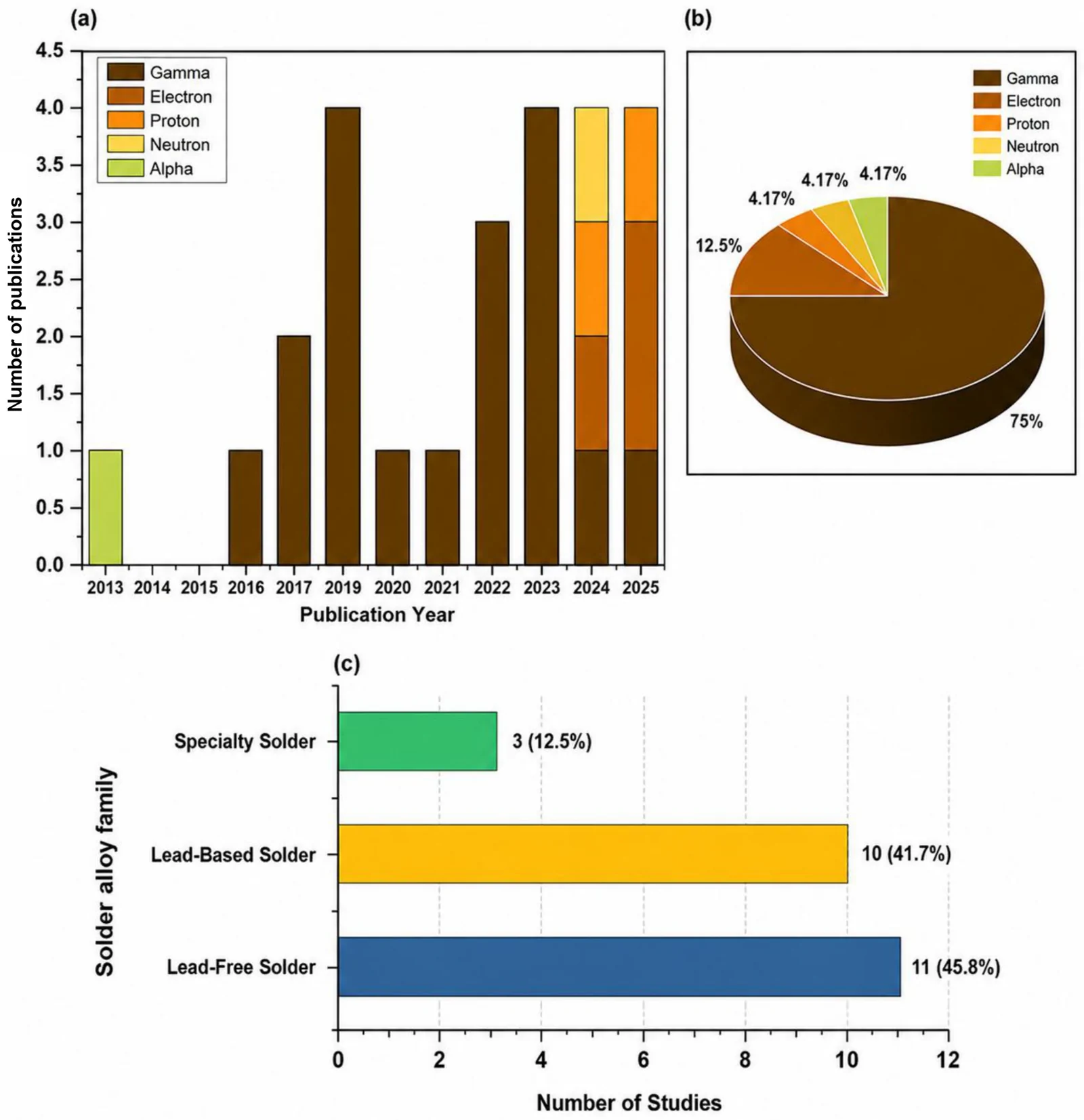
(**a**) Annual publication distribution (2000–2025) of experimental studies on radiation effects in solder joints (**b**) distribution of included studies by radiation type and (**c**) distribution of studies by solder alloy family.

**Figure 3:**
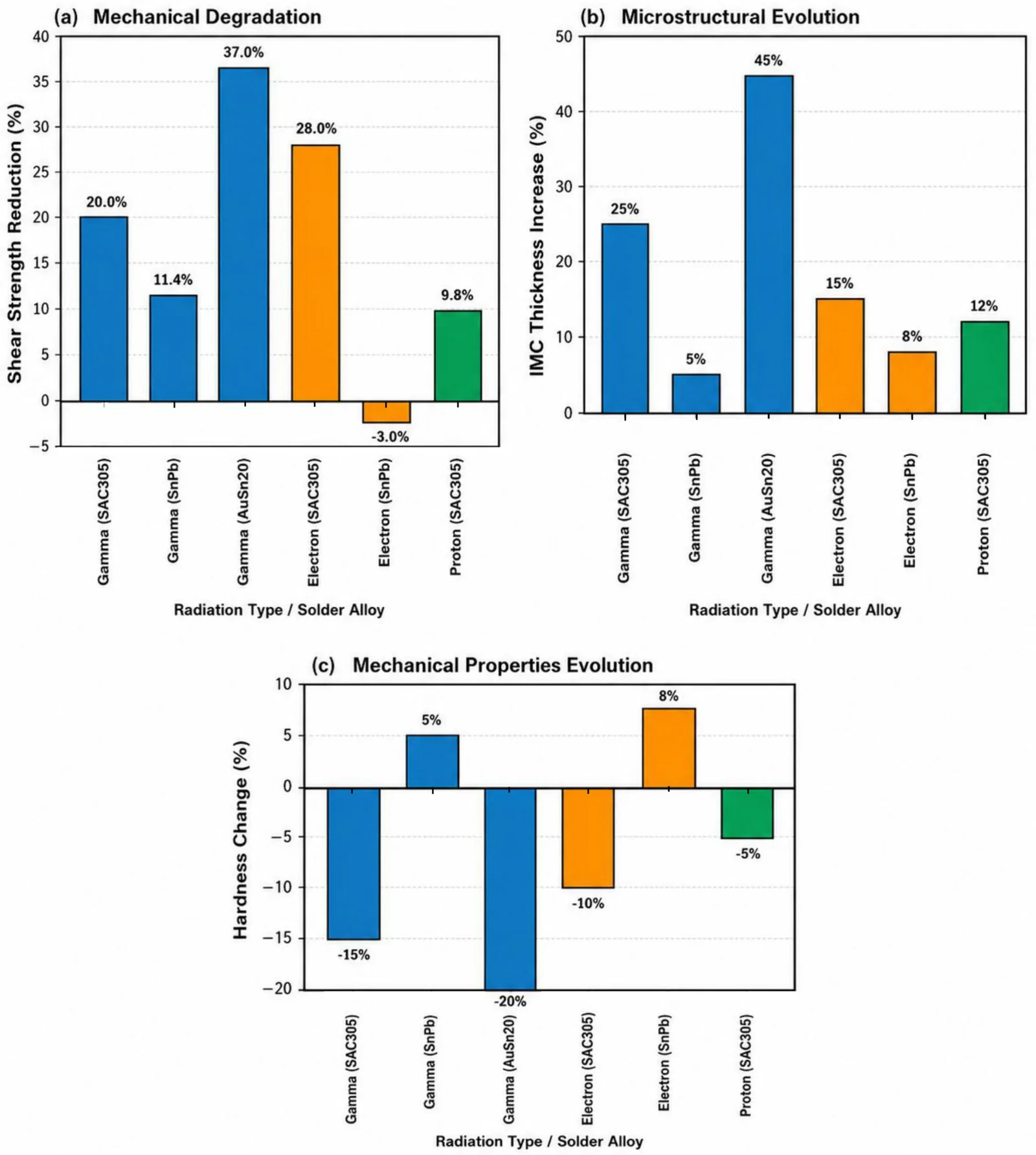
Quantitative comparison of radiation-induced degradation in selected solder systems: (**a**) shear strength reduction, (**b**) IMC thickness increase, and (**c**) hardness variation under different radiation types [5,6,7,8,10,19,20,21,22,23].

**Figure 4:**
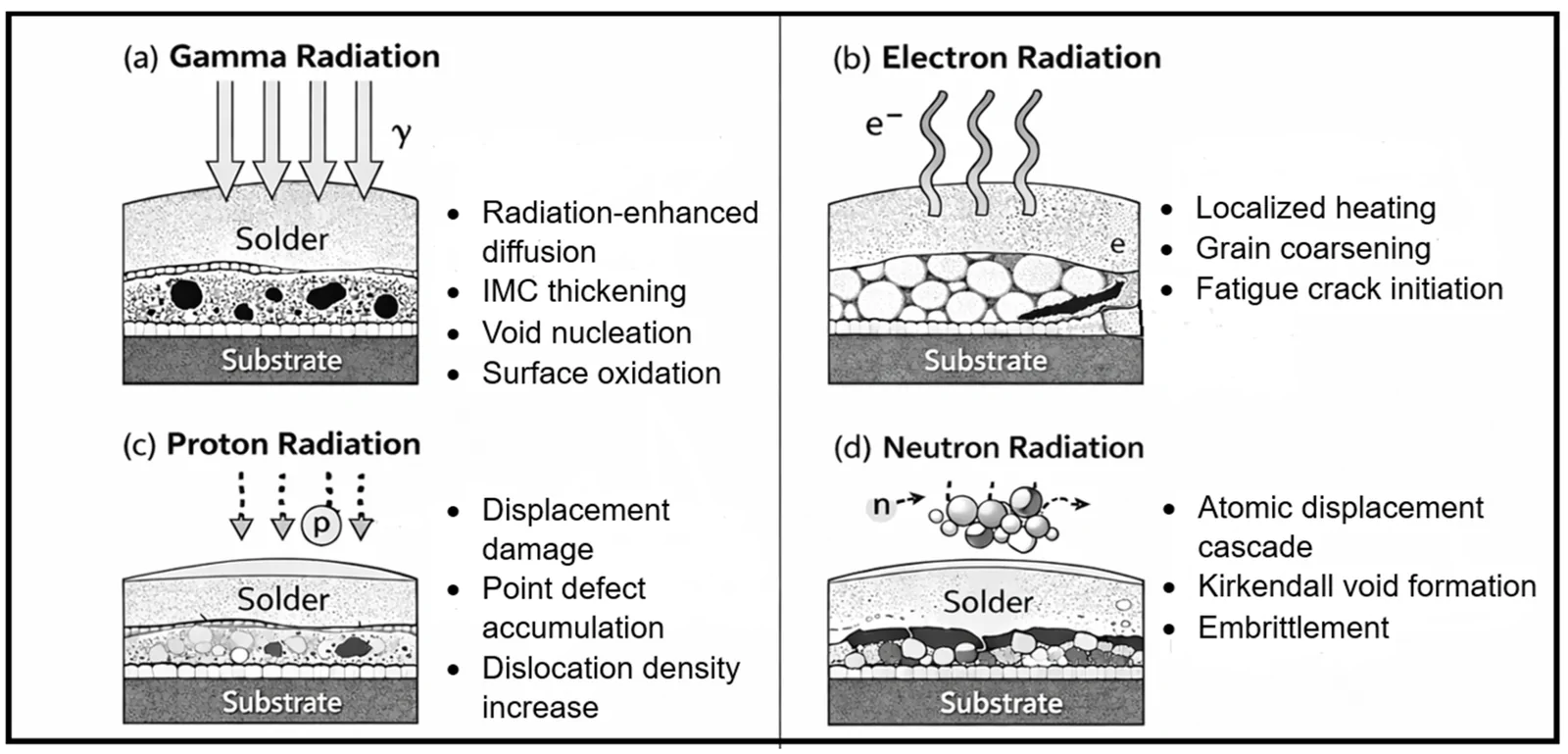
Schematic illustration of radiation-induced degradation mechanisms in solder joints under different irradiation types [8,11,23,31,32,37].

**Figure 5:**
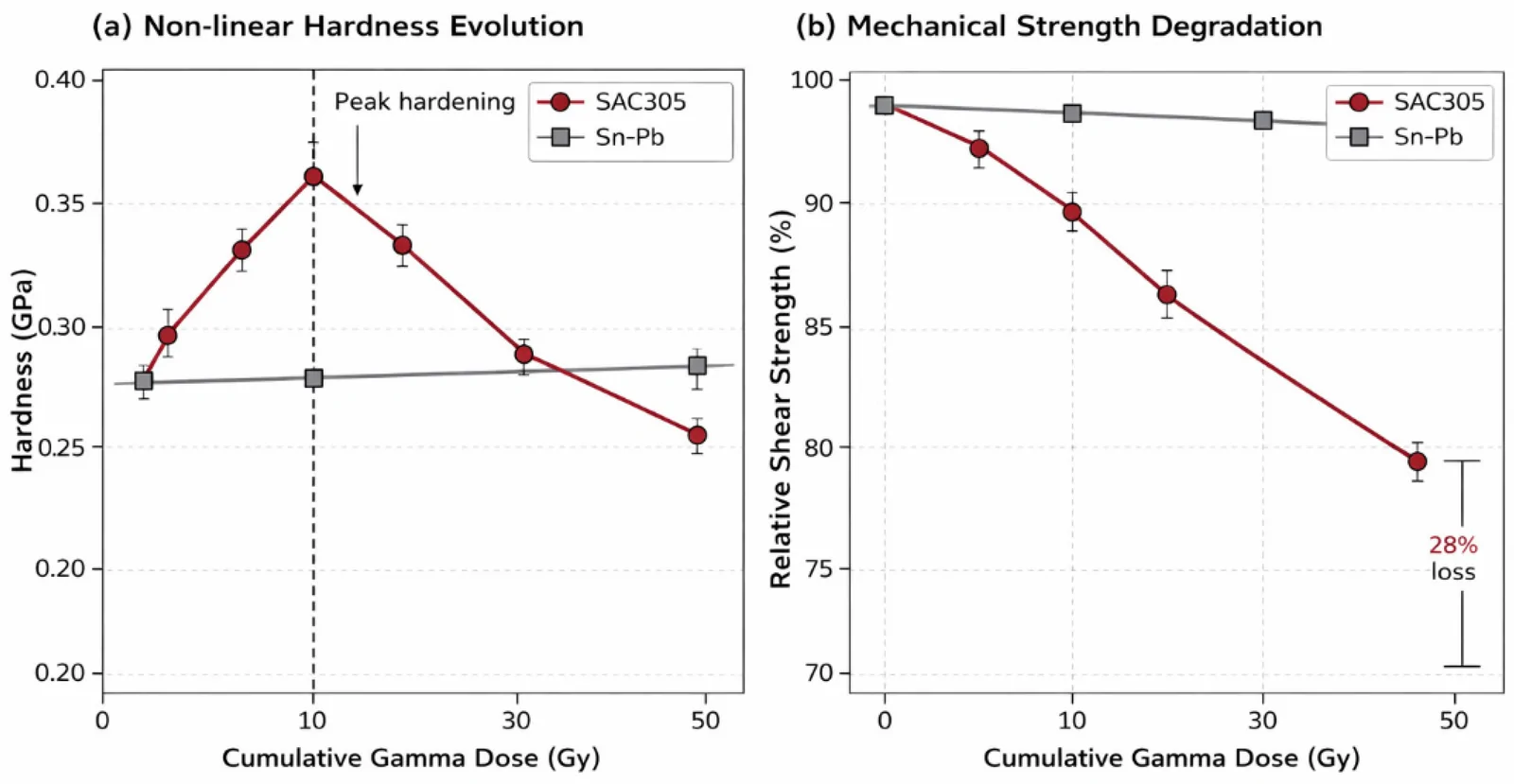
Representative dose-dependent mechanical responses reported in irradiated solder joints. (**a**) Non-linear hardness evolution observed in nanoindentation studies, and (**b**) reduction in relative shear strength under increasing gamma irradiation dose [5,7,8,9,27,28].

**Figure 6:**
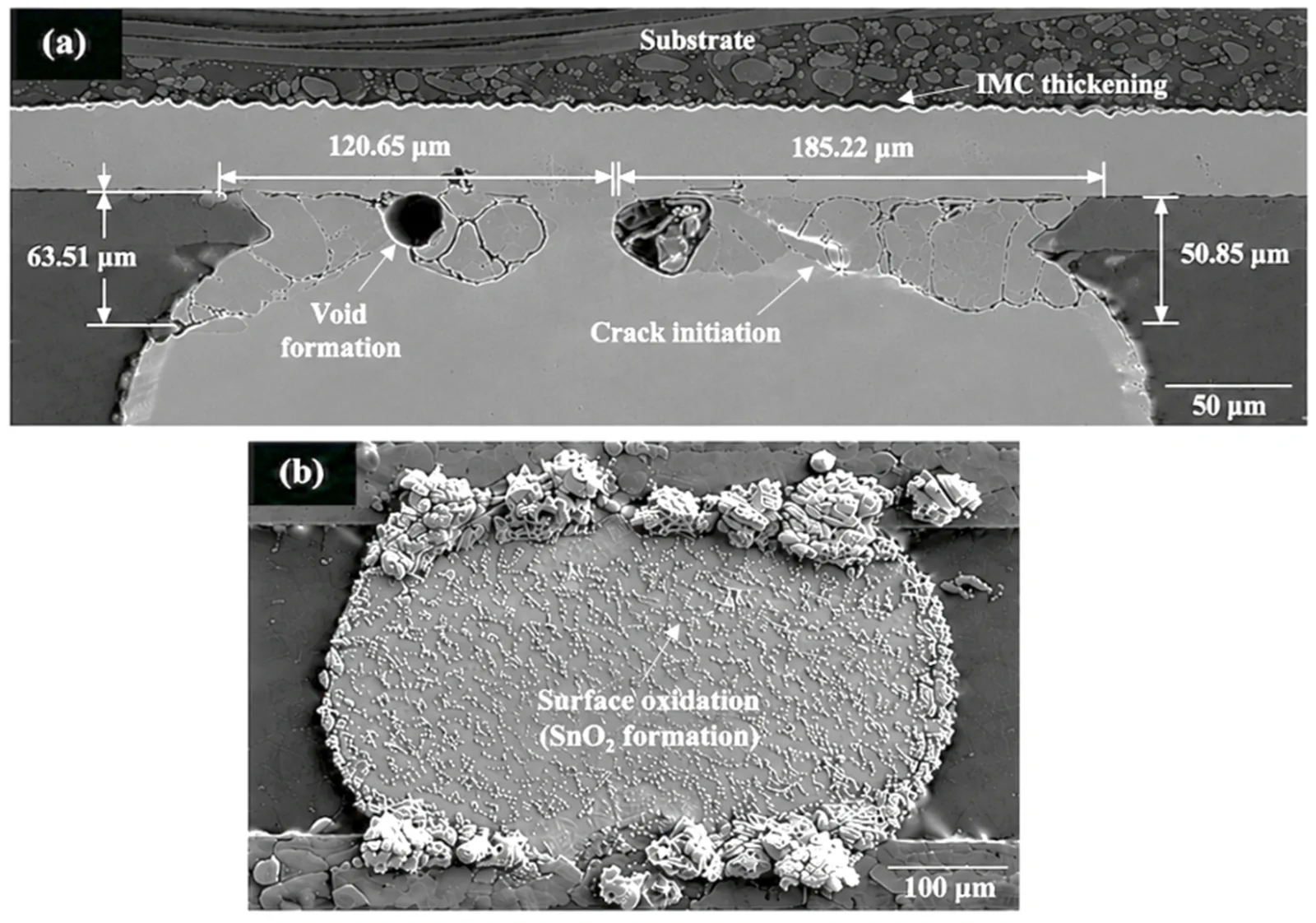
Representative SEM observations of radiation-induced degradation in solder joints reported in the reviewed studies: (**a**) IMC layer thickening, void formation and crack initiation (**b**) surface oxidation following irradiation [8].

**Figure 7:**
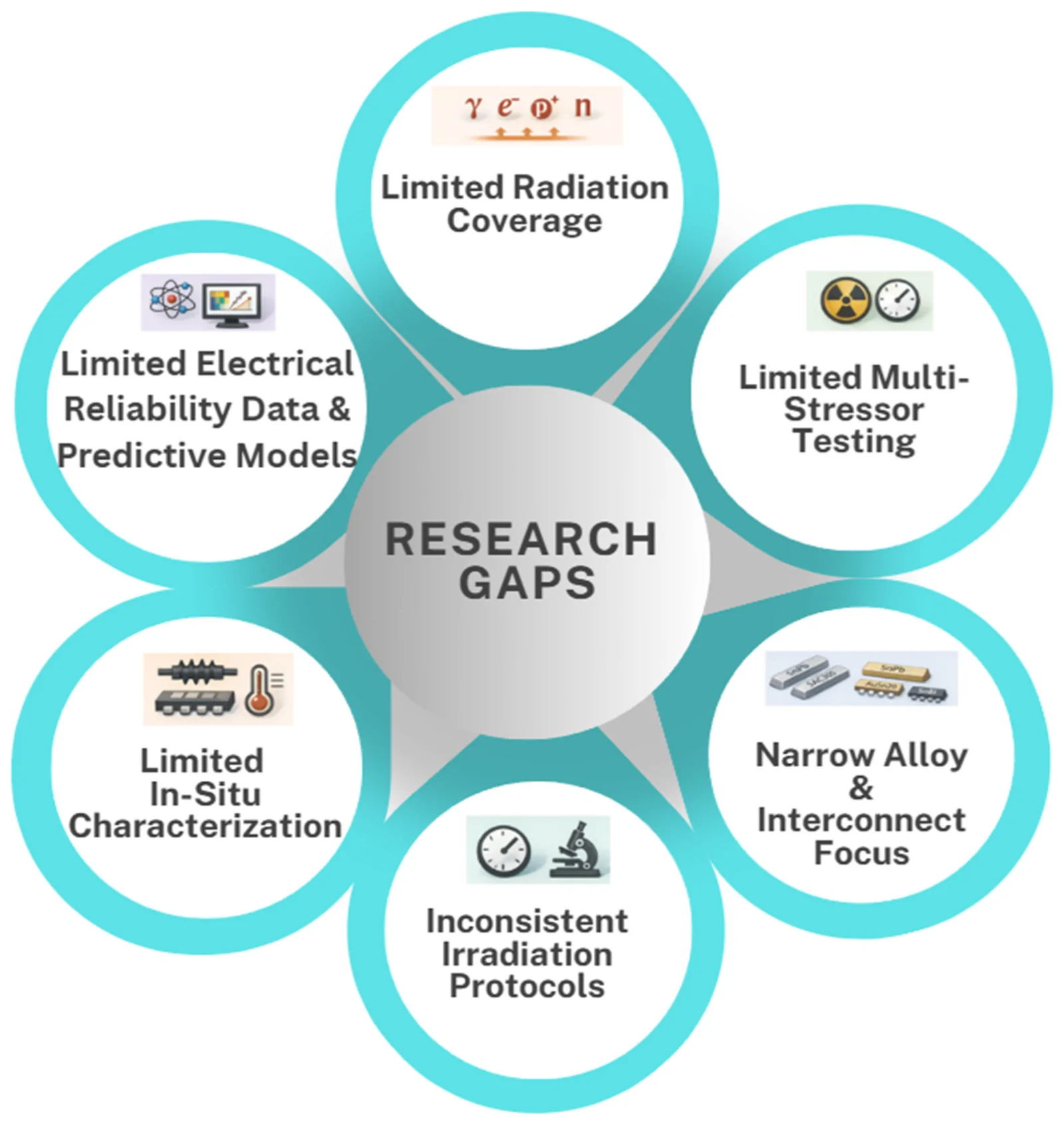
Summary of the key research gaps identified from the reviewed studies on radiation effects in solder joints. The major gaps include limited radiation coverage, limited multi-stressor testing, narrow alloy and interconnect focus, inconsistent irradiation protocols, limited in situ characterization, and limited electrical reliability data and predictive models [1,11,23,32,57,58].

**Table 1:** Search string.

Web of Science	TI = (“solder joint” OR “solder alloy” OR “lead-free solder” OR SnAgCu OR SAC305 OR “SnPb” OR AuSn OR SnBi) AND TS = ((“radiation effect*” OR irradiation OR “ionizing radiation”) NEAR/5 (reliabilit* OR degrad* OR failure* OR “intermetallic compound*” OR IMC))
Scopus	“radiation” OR “gamma irradiation” OR “proton irradiation” OR “neutron irradiation” AND “solder joint” OR “SnPb” OR “SAC305” OR “SnAgCu” AND “microstructure” OR “mechanical properties”

Note: Due to differences in database syntax, the Web of Science and Scopus search strings are not identical. However, both were developed using the same conceptual framework (radiation exposure, solder materials, and reliability-related properties) and were validated using key reference papers before the full search was conducted. The asterisk (*) is a wildcard used to search for words with the same root.

**Table 2:** Summary of selected studies on radiation effects on solder joints.

No.	Authors	Year	Title	Radiation Types	Solder Alloys	Energy/Dose	Findings	Test Method
1.	Kumar et al. [19]	2013	Wetting behavior and elastic properties of low alpha SAC105 and pure Sn solder	α-particles	SAC105	-	- Low alpha SAC105 solders reduce errors, wet better, and are softer than normal solder.	Alpha emission measurement, wetting test, SEM, nanoindentation
2.	El-Daly et al. [20]	2016	Gamma-ray induced modifications in a series of Pb-Free Sn-1.0Ag-0.5Cu-X solder alloys used for radiation shielding	Gamma	Sn–1.0Ag–0.5Cu, Sn–1.0Ag–0.5Cu–2 wt% In, Sn–1.0Ag–0.5Cu–2 wt% Bi	200–800 kGy	- High-dose γ-irradiation induced anomalous strain softening behavior. At a critical dose of ~400 kGy, enhanced ductility accompanied by a slight decrease in strength was observed. - Bi-containing solders showed increased yield and ultimate tensile strengths with increasing dose but reduced ductility. - Sn–1.0Ag–0.5Cu and In-containing alloys were only slightly affected at higher doses. - Mechanical property changes were attributed to microscopic deformation mechanisms, including dislocation cell formation and dislocation channel deformation.	Mechanical testing (yield strength, UTS, ductility), structural and microstructural analysis of deformation mechanisms
3.	Wang et al. [21]	2018	Study on the reliability of Sn50Pb49Sb1/Cu solder joints subjected to γ-ray irradiation	Gamma	Sn50Pb49Sb1/Cu	Dose rate: 0.25 Gy (Si)/s Total irradiation time: 960 h	- No obvious change in IMC thickness or morphology at the solder/Cu interface. - Formation and growth of micro-voids and micro-cracks observed in Pb-based solid solution. - Damage attributed to energetic electrons generated by the Compton effect, leading to lattice vacancies and atomic dislocations. - Pull force reduced by ~22% after irradiation. - Fracture remained ductile, but ductility decreased with less pronounced dimples.	Mechanical pull test, microstructural analysis (IMC morphology). fractographic analysis
4.	Wang et al. [7]	2018	Effect of gamma-ray irradiation on microstructure and mechanical property of Sn63Pb37 solder joints	Gamma	Sn63Pb37	60Co γ-ray/0.25 Gy/s	- The irradiated solder joints exhibits a preserved scallop-like morphology of the IMC layer at the interface, and the thickness of the IMC layer remains relatively unchanged compared to the unirradiated solder joint. - The shear force was reduced about 11.4%, and impact toughness, decreased 13.89% after 960 h of irradiation. - Irradiated solder joints exhibit reduced ductility. - Micro-voids and micro-cracks were observed in the solder after the irradiation.	SEM-EDS, XRD, shear test, impact test
5.	Wen et al. [22]	2019	Effects of γ-ray irradiation on microstructure and mechanical property of AuSn20 solder joint	Gamma	AuSn20	60Co γ	- The irradiation increases the number and size of voids in the solder joints. - The irradiation promotes the formation and growth of (Au,Ni)Sn and (Ni,Au)_3_Sn_2_ IMC layers - The failure mode of the solder joint was altered from the ductile fracture to brittle fracture, and the shear strength of joint was decreased by 37.23% after 1000 h γ-ray irradiation.	2D-Xray imaging, SEM-EDS, shear test
6.	Wang et al. [23]	2019	Microstructure and mechanical properties of eutectic SnPb solder joints subjected to γ-ray irradiation	Gamma	SnPb		- Formation of micro-voids and micro-cracks in Pb-rich solid solution due to irradiation damage accumulation. - Tensile force decreased by 14.12% after irradiation. - Fracture remained ductile, but plasticity reduced with less pronounced dimples. - Damage mechanism attributed to energetic electrons generated via Compton effect, leading to point defect formation and vacancy accumulation.	Tensile testing, SEM for microstructure analysis
7.	Wang et al. [24]	2019	Microstructure and performance evolution of SnPbSb solder joint under γ-ray irradiation and thermal cycling	Gamma	SnPbSb	60Co γ-ray/0.25 Gy/s	- The IMC layer thickness of irradiated solder joints are hardly changes. - Micro-voids and micro-cracks were observed in the solder after the irradiation - The strength and ductility of solder joints were decreased after irradiation.	Thermal cycling, shear test, SEM
8.	Wen et al. [12]	2020	Microstructural evolution and shear performance of AuSn20 solder joint under gamma-ray irradiation and thermal cycling	Gamma	Au–20Sn (AuSn20) solder joint	γ-ray irradiation time: 1000 h; Thermal cycling: 200 cycles	- Shear force of AuSn20 micro-joints decreased by 17.5% after γ-ray irradiation and by 25.3% after thermal cycling. - Degradation attributed to increased thickness of interfacial IMC layers and void formation. - Both radiation and thermal cycling negatively affected joint reliability, with thermal cycling having a more pronounced impact. - Results highlight reliability concerns of AuSn20 solder joints in space environments without RHBD or active thermal control systems.	Shear testing of micro-joints, microstructural analysis (IMC layer growth, void observation)
9.	Rahmat et al. [25]	2021	Effect of gamma radiation on micromechanical behaviour of SnPb solder alloy	Gamma	SnPb	60Co γ-ray/0.25 Gy/s	- Mechanical properties changed due to the irradiation - Hardness decrease - Reduced modulus increase	Nanoindentation
10.	Mohammed et al. [26]	2022	Enhancing the tensile properties of Sn-Zn-Ag lead-free solder alloy by loading MgO nanoparticles and irradiation	Gamma	Sn–5.3 wt% Zn–1 wt% Ag (SZA531) and SZA531/0.5 wt% MgO nanocomposite	0–2.0 MGy Tested at various strain rates (ε•)	- MgO nanoparticles (~9.61 nm) were pure and crystalline. Both plain and composite solders consisted of β-Sn, Zn, Ag, and IMCs (Ag_3_Sn, AgZn). MgO loading refined β-Sn and Ag_3_Sn phases and partially dissolved AgZn. - Melting point slightly increased (206.1 °C → 208.7 °C), indicating good solderability. - Ultimate tensile strength and tensile toughness increased with higher strain rate, γ-dose, and MgO loading, while ductility decreased. - Strain-rate sensitivity index (m) decreased with increasing dose and MgO due to reduced resistance to necking. - Strengthening attributed to Orowan and dislocation strengthening mechanisms.	Tensile testing at different strain rates, microstructural analysis (phase identification, morphology), thermal analysis (melting point)
11.	Lehan et al. [27]	2022	EDS analysis on effect of low dosage gamma radiation and micromechanical properties of SnAg3Cu0.5 solder	Gamma	SAC305	60Co γ-ray/0.25 Gy/s	-Higher radiation dose made the solder softer and more ductile. -FESEM and EDS showed intermetallics were mainly Cu_6_Sn_5_ and Ag_2_Sn. -Hardness and reduced modulus decreased with increasing radiation dose. -The reduction was due to microstructure changes and void formation.	Nanoindentation, FESEM EDS, optical microscope
12.	Lehan et al. [28]	2022	Influence of gamma radiation on eutectic phase area and hardness properties of SAC305 solder	Gamma	SAC305	60Co γ-ray/0.23 Gy/s	-Gamma radiation increased IMC thickness and altered the eutectic phase area, peaking at 29.65% at 15 Gy before dropping to 17.66% at 25 Gy -Hardness rose to 0.36 GPa with radiation up to 15 Gy, then declined to 0.23 GPa at higher doses. -Structural and atomic changes from gamma radiation, including heat-induced coarsening, influenced both microstructure and hardness.	Optical microscope, nanoindentation
13.	Guan et al. [8]	2023	Effect of gamma irradiation on microstructural evolution and mechanical properties of Sn3Ag0.5Cu solder joints	Gamma	SAC305	60Co γ-ray/1 Gy/s	- The gamma radiation induces the oxidation of solder joint. - Micro-voids and micro-cracks were formed after irradiation. - Hardness exhibited a non-monotonic response, increasing from 0.244 GPa (0 h) to 0.271 GPa (50 h), decreasing to 0.259 GPa (100 h), and subsequently rising to 0.273 GPa (200 h).	DSC, SEM EDS, HRTEM, nanoindentation
14.	Guan et al. [9]	2023	Effect of gamma irradiation on microstructural evolution and mechanical properties of SnPb eutectic solder joints	Gamma	SnPb	60Co γ-ray/1 Gy/s	Irradiation accelerated the oxidation of Sn–Pb eutectic solder joints. - Irradiation induced the nucleation and growth of SnO2 nanocrystals and Sn wrinkles in the Sn-based solid solution. - Generation of Sn and Pb depletion holes on the solder joint surface due to irradiation. - The depletion of Sn was more severe than that of Pb. - Shear force increased from 375.64 g to 408.88 g after 100 h irradiation, followed by a slight decrease to 395.87 g after 200 h.	DSC, SEM EDS, TEM, XPS, shear test
15.	Ismail et al. [10]	2024	Microstructural evolution of 96.5Sn-3.0Ag-0.5Cu (SAC305) solder joints induced by variation doses of gamma-irradiation	Gamma	SAC305	0.25 Gy/s	-Increased β-Sn and Ag_3_Sn intensities with higher gamma doses, no new phases formed. -Eutectic phase peaked at 15 Gy, then decreased; IMC thickness increased with dose. -Cracks appeared near the IMC-solder interface under high radiation, affecting reliability.	Optical microscope, FESEM EDS, XRD, nanoindentation
16.	Wan Yusoff et al. [5]	2023	Micromechanical response of SAC305 solder alloy under gamma radiation via nanoindentation approach	Gamma	SAC305	5–50 Gy	- Hardness of SAC305 increased with dose up to 15 Gy, reaching a maximum of 0.37 GPa, attributed to high defect density induced by γ-irradiation. - At higher doses (15–50 Gy), hardness decreased to 0.13 GPa, indicating radiation-induced softening due to defect size evolution. - Creep behaviour was significantly affected by irradiation; creep rates increased at doses from 10 Gy to 50 Gy. - Gamma irradiation caused SAC305 solder to become more ductile compared to the non-irradiated alloy. - Stress exponent analysis suggested different deformation mechanisms operating at different gamma doses.	Nanoindentation testing (hardness and creep); Micromechanical analysis of deformation mechanisms
17.	Liu et al. [29]	2024	Effect of electron irradiation on microstructural evolution and mechanical properties of Sn3Ag0.5Cu solder joints	Proton, Electron	SAC305	Proton: 1 MeV, fluence of 1 × 10^14^ p/cm^2^ - Electron: 1 MeV, fluence of 1 × 10^14^ e/cm^2^	- Radiation (both types) led to grain coarsening and crack formation in solder joints - Degradation of tensile strength after radiation exposure - Formation of Kirkendall voids and microstructural damage more significant under proton than electron irradiation	SEM EDS, tensile testing room temperature
18.	Jing et al. [30]	2024	Effects of neutron irradiation on failure behavior analysis of high-density bumps under thermo-coupling conditions	Neutron	SnAg	-	- Neutron irradiation causes atomic vacancies and defect clusters, enhancing the Kirkendall effect - Defects hinder slip dislocation, increasing hardness and elastic modulus - Excessive defects lead to bump failure	SEM, EBSD, nanoindentation, push ball experiment
19.	Wu et al. [31]	2024	The comprehensive effect of electronic irradiation and thermal cycling on the thermal and mechanical properties of Sn-3.0Ag-0.5Cu solder joints	Electron	SAC305	100 Mrad (Si) electron dose + 200 thermal cycles	- Electron irradiation and thermal cycling together degrade solder joint performance - Shear strength decreased by 28.00% (brittle) and 23.34% (plastic) - Resistor temperature increased - Plastic fracture becomes more dominant with increased cycling	- Shear test, electrical performance monitoring (resistor temperature), fractography
20.	Guan et al. [32]	2024	Pb segregation in SnPb eutectic solder alloy induced by gamma irradiation	Gamma	SnPb eutectic solder joint	Irradiation time: 100–150 h	- Pb segregation occurred after 100 h of γ-ray irradiation, forming a distinct Pb-rich segregation area in the solder joint. - Pb segregation ceased after prolonged irradiation (150 h). The segregation mechanism was attributed to γ-ray–induced oxidation of β-Sn to SnO_2_, which drove Pb enrichment. - Predicted Pb content in the segregation area (53.5%) based on segregation heat formulas agreed well with experimental measurement (49.11%). - DFT and MD simulations confirmed that Pb segregation was driven by β-Sn oxidation under γ-irradiation.	Microstructural and compositional analysis (Pb segregation observation); theoretical analysis (segregation heat model); Density Functional Theory (DFT) calculation; Molecular Dynamics (MD) simulation
21.	Liu et al. [33]	2025	Effects of proton irradiation on microstructure and mechanical properties of SAC305 solder joints	Proton	SAC305	30 MeV protons, fluence of 1 × 10^11^ p/cm^2^	- Protons caused nuclear reactions, increasing dislocation density and inducing lattice defects - Grain size reduced at nanoscale - No visible cracks or voids, but decreased lattice quality - Shear strength dropped by 9.8%	Shear test, SEM, Simulation to study proton-solder interaction
22.	Guan et al. [34]	2025	Effect of electron irradiation on the mechanical properties of SnPb eutectic solder joints: A combined experimental and first-principles study	Electron	SnPb eutectic solder	1.2 MeV electrons; fluence of 1 × 10^16^ to 2 × 10^16^ e/cm^2^	- Shear strength initially increases to 48.35 MPa at 1 × 10^16^ e/cm^2^ due to irradiation-induced electron localization, then decreases to 47.32 MPa at 2 × 10^16^ e/cm^2^ with reduced ductility. - Fracture occurs in the bulk solder region - Defective Sn/Pb interfaces show higher shear strength than ideal interfaces.	Experimental shear strength testing of solder joints combined with first principles (DFT) calculations and displacement–energy curve fitting
23.	Guan et al. [35]	2025	Interface-driven anomalies in irradiation damage: Multiscale simulation of asymmetric diffusion and mechanical degradation in SnPb solder joints under electron irradiation	Electron irradiation	SnPb eutectic solder	1.2 MeV electrons (fluence not explicitly stated; irradiation-dependent evolution observed)	- Reveals asymmetric diffusion where Sn atoms show enhanced mobility into Pb-rich regions, causing interfacial instability and accelerated microstructural degradation. - Sn phase exhibits higher susceptibility to irradiation-induced amorphization than Pb, indicating phase-dependent degradation. - Mechanical behavior shows initial irradiation-induced strengthening followed by ductility degradation due to microvoid and microcrack formation. -Irradiation-induced defects alter Sn/Pb interfacial bonding and shear response.	Multiscale approach combining experimental microstructural characterization and mechanical testing (shear behavior, fracture analysis) with DFT calculations of interfacial shear and bonding
24.	Guan et al. [36]	2025	Mechanisms of γ-ray irradiation induced oxidation corrosion in SnBi eutectic solder joints: Insights from first-principles calculations	Gamma	SnBi eutectic solder	γ-ray exposure up to 200 h (dose rate not specified)	- Preferential formation of Sn oxides at the Sn/Bi interface; with prolonged irradiation, both Sn and Bi oxides accumulate, and the solder surface becomes fully covered by Sn oxides after 200 h. - γ-ray–induced atomic vacancies enhance O_2_ adsorption and accelerate oxidation. - Bi incorporation lowers the work function of Sn-based solid solutions, increasing Sn’s susceptibility to oxidation. - Low diffusion energy barrier of Sn through Sn/SnO_2_ promotes continuous SnO_2_ layer thickening.	Quasi-in situ microstructural and surface characterization (oxidation evolution) combined with first principles (DFT) calculations on vacancy formation, O_2_ adsorption, work function, and diffusion behavior

**Table 3:** Summary of radiation-type-dependent degradation behavior in solder joints.

Radiation Type	Solder Alloys	Dominant Degradation Mechanisms	Representative Properties Changes	Typical Dose/Fluence Range
Gamma (γ)	SAC305	IMC thickening (Ag_3_Sn, Cu_6_Sn_5_), eutectic instability, oxidation, microvoids	Hardness: ↑ at ≤15 Gy then ↓; Shear strength: −20% to −37%	5–800 kGy; 5–50 Gy; up to 1000 h
	SnPb	Sn oxidation (SnO_2_), Pb segregation, microvoids	Shear strength: −10% to −22%; transient strengthening then softening	0.25–1 Gy/s; 100–960 h
	AuSn20	Excessive IMC growth, void coalescence, brittle fracture	Shear strength: −17% to −37%	Up to 1000 h
	SnBi	Oxidation corrosion, SnO_2_ layer thickening	Surface embrittlement; strength degradation	Up to 200 h
Electron	SAC305	Grain coarsening, microcracks, defect accumulation	Shear strength: −23% to −28%; ↑ resistance	~100 Mrad (Si)
	SnPb	Interfacial instability, asymmetric Sn/Pb diffusion	Shear strength: ↑ at low fluence, ↓ at high fluence	1.2 MeV; 10^16^ e/cm^2^
Proton	SAC305	Displacement damage, lattice disorder, dislocation accumulation	Shear strength: −9% to −10%; no visible cracks	1–30 MeV; 10^11^–10^14^ p/cm^2^
Neutron	SnAg	Vacancy clusters, Kirkendall voiding, brittle stiffening	Hardness & modulus ↑; premature failure risk	Not reported
Alpha	SAC105	Reduced alpha emission, refined microstructure	Improved wetting; reduced soft error risk	Low-alpha emission

**Note:** ↑ = increase; ↓ = decrease. The values summarized in Table 3 represent general trends reported across the reviewed studies and should be interpreted with caution due to differences in radiation conditions, solder alloys, and experimental methods. Dose unit conversion: 1 Mrad = 10 kGy; values originally reported in Mrad have been converted to kGy for consistency.

## Data Availability

No new data were created or analyzed in this study. Data sharing is not applicable to this article.

## Supplementary Materials

The following supporting information can be downloaded at: https://www.mdpi.com/article/10.3390/ma19194175/s1, PRISMA 2020 checklist. Ref. [18] is included in the Supplementary Materials.
