# Supplementary material for: Radiation-Induced Interfacial Degradation and Mechanical Reliability of Sn-Based Solder Joints: A Systematic Review
Source: Materials (Basel). 2026 Sep 30;19(19):4175. doi: 10.3390/ma19194175 (PMC13666471; doi:10.3390/ma19194175)
Supplement: Supplementary file 1 [file materials-19-04175-s001.zip › materials-4351174-supplementary.pdf]

PRISMA 2020 Checklist

Section and Topic	Item #	Checklist item	Location where item is reported
**TITLE**			
Title	1	Identify the report as a systematic review.	Title page – "Radiation-Induced Interfacial Degradation and Mechanical Reliability of Sn-Based Solder Joints: A Systematic Review"
**ABSTRACT**			
Abstract	2	See the PRISMA 2020 for Abstracts checklist.	Abstract (pp. 1–2) – structured abstract covering background, methods, results, conclusions
**INTRODUCTION**			
Rationale	3	Describe the rationale for the review in the context of existing knowledge.	Section 1 (Introduction), paragraphs 1–4 – rationale for reviewing radiation effects on Sn-based solder joints given gap in mechanistic understanding
Objectives	4	Provide an explicit statement of the objective(s) or question(s) the review addresses.	Section 2.1 (Research Questions) – five explicit RQs (RQ1–RQ5) on radiation types, microstructural/mechanical/electrical effects, alloy tolerance, experimental conditions, and research gaps
**METHODS**			
Eligibility criteria	5	Specify the inclusion and exclusion criteria for the review and how studies were grouped for the syntheses.	Section 2.3 (Inclusion and Exclusion Criteria) – peer-reviewed journal articles, 2000–2025, English, radiation-effects on solder joints; excluded: non-journal, no solder focus, no full text, insufficient technical detail
Information sources	6	Specify all databases, registers, websites, organisations, reference lists and other sources searched or consulted to identify studies. Specify the date when each source was last searched or consulted.	Section 2.2 (Literature Search Strategy) – Web of Science and Scopus; search period 2000–2025; Table 1 shows full Boolean strings; last searched prior to manuscript submission
Search strategy	7	Present the full search strategies for all databases, registers and websites, including any filters and limits used.	Section 2.2 (Literature Search Strategy), Table 1 – full Boolean search strings for Web of Science (TI/TS fields, NEAR/5 operator) and Scopus (TITLE-ABS-KEY)
Selection process	8	Specify the methods used to decide whether a study met the inclusion criteria of the review, including how many reviewers screened each record and each report retrieved, whether they worked independently, and if applicable, details	Section 2.4 (Study Selection Process) – two-database search, year/language/document-type filter,

PRISMA 2020 Checklist

Section and Topic	Item #	Checklist item	Location where item is reported
		of automation tools used in the process.	duplicate removal, title-and-abstract screening by authors; consensus-based inclusion; PRISMA flow diagram (Figure 1)
Data collection process	9	Specify the methods used to collect data from reports, including how many reviewers collected data from each report, whether they worked independently, any processes for obtaining or confirming data from study investigators, and if applicable, details of automation tools used in the process.	Section 2.5 (Data Extraction and Synthesis) – structured extraction table completed by authors; items: authorship, year, radiation type, alloy, dose/energy/duration, outcomes (microstructure, mechanical, electrical), techniques
Data items	10a	List and define all outcomes for which data were sought. Specify whether all results that were compatible with each outcome domain in each study were sought (e.g. for all measures, time points, analyses), and if not, the methods used to decide which results to collect.	Section 2.5 (Data Extraction and Synthesis) – outcomes: microstructural evolution, mechanical strength/hardness/ductility, electrical resistance, thermal behaviour; all reported results extracted per study
	10b	List and define all other variables for which data were sought (e.g. participant and intervention characteristics, funding sources). Describe any assumptions made about any missing or unclear information.	Section 2.5 (Data Extraction and Synthesis) – variables: radiation type, solder alloy composition, dose/fluence/energy/duration, exposure environment, characterisation techniques (SEM, EDS, XRD, nanoindentation, shear/tensile testing)
Study risk of bias assessment	11	Specify the methods used to assess risk of bias in the included studies, including details of the tool(s) used, how many reviewers assessed each study and whether they worked independently, and if applicable, details of automation tools used in the process.	Not applicable – this SLR is qualitative/narrative; no formal risk-of-bias tool (e.g. RoB 2) was applied. Study quality was assessed through the inclusion criteria (peer-reviewed journals, adequate technical detail) as described in Section 2.3
Effect measures	12	Specify for each outcome the effect measure(s) (e.g. risk ratio, mean difference) used in the synthesis or presentation of results.	Not applicable – no meta-analysis was performed. Results are synthesised qualitatively; quantitative ranges (e.g. shear strength reductions) are reported descriptively in Sections 3.2.1–3.2.4 and Table 3
Synthesis methods	13a	Describe the processes used to decide which studies were eligible for each synthesis (e.g. tabulating the study intervention characteristics and comparing against the planned groups for each synthesis (item #5)).	Section 2.5 (Data Extraction and Synthesis) – studies grouped by radiation type and solder alloy

PRISMA 2020 Checklist

Section and Topic	Item #	Checklist item	Location where item is reported
			composition for thematic synthesis; grouping criteria consistent with RQ1–RQ3 and eligibility criteria (Section 2.3)
	13b	Describe any methods required to prepare the data for presentation or synthesis, such as handling of missing summary statistics, or data conversions.	Section 2.5 – no statistical data transformation required; quantitative values (e.g. % shear-strength reduction, IMC thickness) reported as extracted from individual studies; ranges noted where multiple studies reported the same metric
	13c	Describe any methods used to tabulate or visually display results of individual studies and syntheses.	Sections 3.1–3.2 and Figures 3–5 – results tabulated in Table 2 (individual studies) and Table 3 (grouped by radiation type); bar/spider/heat-map charts (Figures 2–5) visually display comparative degradation data
	13d	Describe any methods used to synthesize results and provide a rationale for the choice(s). If meta-analysis was performed, describe the model(s), method(s) to identify the presence and extent of statistical heterogeneity, and software package(s) used.	Section 2.5 (Data Extraction and Synthesis) and Section 4 (Discussion) – qualitative narrative synthesis chosen because heterogeneous experimental conditions precluded meta-analysis; grouped by radiation type and alloy; RED framework integrates findings (Section 4.4)
	13e	Describe any methods used to explore possible causes of heterogeneity among study results (e.g. subgroup analysis, meta-regression).	Sections 3.2.1–3.2.2 – radiation type and alloy composition explored as sources of heterogeneity; no formal meta-regression conducted; alloy-dependent and dose-dependent differences discussed qualitatively in Sections 3.2.2 and 3.2.4
	13f	Describe any sensitivity analyses conducted to assess robustness of the synthesized results.	Not applicable – no formal sensitivity analysis performed; qualitative robustness assessed by cross-referencing findings across multiple independent studies for each radiation type and alloy (Sections 3.2.1–3.2.4)
Reporting bias assessment	14	Describe any methods used to assess risk of bias due to missing results in a synthesis (arising from reporting biases).	Section 3.2.6 (Research Gaps and Limitations) and Figure 8 – methodological inconsistencies and

PRISMA 2020 Checklist

Section and Topic	Item #	Checklist item	Location where item is reported
			underrepresentation of radiation types noted; no funnel plot or formal small-study-effects test conducted given qualitative synthesis
Certainty assessment	15	Describe any methods used to assess certainty (or confidence) in the body of evidence for an outcome.	Not applicable – no formal GRADE or equivalent certainty assessment performed; evidence quality addressed narratively through discussion of methodological limitations in Section 3.2.6 and Figure 8
**RESULTS**			
Study selection	16a	Describe the results of the search and selection process, from the number of records identified in the search to the number of studies included in the review, ideally using a flow diagram.	Section 2.4 (Study Selection Process) and Figure 1 (PRISMA flow diagram) – 98 records identified (WoS: 46, Scopus: 52); 66 after year/language/document-type filter; 8 duplicates removed; 58 screened; 24 included
	16b	Cite studies that might appear to meet the inclusion criteria, but which were excluded, and explain why they were excluded.	Section 2.4 – studies excluded at full-text review stage for not meeting inclusion criteria (no direct radiation-on-solder-joint focus, insufficient technical detail, no full-text access) are described; no separate excluded-study list provided beyond Figure 1
Study characteristics	17	Cite each included study and present its characteristics.	Section 3.1 (Background of Selected Articles) and Table 2 – all 24 included studies cited with author, year, title, radiation type, alloy, dose/energy, key findings, and test methods
Risk of bias in studies	18	Present assessments of risk of bias for each included study.	Not applicable – formal risk-of-bias assessment per included study was not conducted; see Section 3.2.6 for discussion of methodological limitations across studies
Results of individual studies	19	For all outcomes, present, for each study: (a) summary statistics for each group (where appropriate) and (b) an effect estimate and its precision (e.g. confidence/credible interval), ideally using structured tables or plots.	Table 2 (Section 3.1) – per-study summary of key quantitative and qualitative findings; Figure 3 presents comparative mechanical degradation (shear strength, IMC thickness, hardness) across studies

PRISMA 2020 Checklist

Section and Topic	Item #	Checklist item	Location where item is reported
			and radiation types
Results of syntheses	20a	For each synthesis, briefly summarise the characteristics and risk of bias among contributing studies.	Sections 3.2.1–3.2.4 – for each thematic synthesis (by radiation type and alloy), contributing studies' characteristics and limitations are summarised; Figure 5 heat map aggregates alloy performance ratings
	20b	Present results of all statistical syntheses conducted. If meta-analysis was done, present for each the summary estimate and its precision (e.g. confidence/credible interval) and measures of statistical heterogeneity. If comparing groups, describe the direction of the effect.	Not applicable – no meta-analysis was performed; quantitative ranges synthesised narratively in Sections 3.2.1, 3.2.2, 3.2.4, and Table 3 (e.g. shear strength reductions 11–37%, IMC growth thresholds)
	20c	Present results of all investigations of possible causes of heterogeneity among study results.	Sections 3.2.1–3.2.2 – radiation type and alloy composition identified as key sources of heterogeneity; dose-dependent non-linearity explored in Section 3.2.4 and Figure 7; no formal meta-regression
	20d	Present results of all sensitivity analyses conducted to assess the robustness of the synthesized results.	Not applicable – no formal sensitivity analyses conducted; robustness of narrative conclusions assessed by consistency of findings across multiple studies per radiation type (Sections 3.2.1–3.2.4)
Reporting biases	21	Present assessments of risk of bias due to missing results (arising from reporting biases) for each synthesis assessed.	Section 3.2.6 and Figure 8 – reporting biases discussed qualitatively; predominance of gamma-irradiation studies and limited neutron/proton data noted as potential source of publication bias
Certainty of evidence	22	Present assessments of certainty (or confidence) in the body of evidence for each outcome assessed.	Not applicable – no formal GRADE certainty assessment; evidence limitations discussed in Section 3.2.6 (Figure 8) including lack of standardised protocols, limited multi-stressor data, and sparse electrical/thermal characterisation
**DISCUSSION**			
Discussion	23a	Provide a general interpretation of the results in the context of other evidence.	Section 4 (Discussion), Sections 4.1–4.5 – findings interpreted in context of radiation materials science, RED framework (Section

Section and Topic	Item #	Checklist item	Location where item is reported
			4.4), and existing reviews; comparisons with prior narrative reviews including authors' earlier work [16]
	23b	Discuss any limitations of the evidence included in the review.	Section 3.2.6 (Research Gaps and Limitations) and Figure 8 – limitations of included evidence: heterogeneous irradiation conditions, lack of standardised protocols, limited neutron/proton data, sparse electrical/thermal characterisation
	23c	Discuss any limitations of the review processes used.	Section 3.2.6 and Section 4.5 – review limitations: qualitative synthesis only, no formal bias assessment, concentration on SnPb/SAC305, post-irradiation-only characterisation, absence of in-situ methods
	23d	Discuss implications of the results for practice, policy, and future research.	Section 4.5 (Synthesis and Path Forward) and Section 5 (Conclusion) – implications for radiation-aware alloy design, qualification protocols (multi-stressor, radiation-type-specific), and future research priorities (neutron database, electrical metrics, predictive modelling)
**OTHER INFORMATION**			
Registration and protocol	24a	Provide registration information for the review, including register name and registration number, or state that the review was not registered.	Not registered – no systematic review registration number is reported; the authors state this is a PRISMA-compliant SLR but do not cite a registration in PROSPERO or equivalent registry
	24b	Indicate where the review protocol can be accessed, or state that a protocol was not prepared.	No protocol document prepared or publicly archived; methodology described in full in Section 2 (Methodology)
	24c	Describe and explain any amendments to information provided at registration or in the protocol.	Not applicable – no prior registration or protocol; no amendments to report
Support	25	Describe sources of financial or non-financial support for the review, and the role of the funders or sponsors in the review.	Acknowledgments section – funded by FRGS Grant No.

PRISMA 2020 Checklist

Section and Topic	Item #	Checklist item	Location where item is reported
			FRGS/1/2022/TK04/UNIKL/02/12 (Ministry of Higher Education Malaysia / UniKL MIAT) and UPNM PPPI Grant No. PS060-UPNM/2023/GPPP/SG/1; funders had no role in study design, data collection, or reporting
Competing interests	26	Declare any competing interests of review authors.	Declaration of Competing Interest – "The authors declare that they have no known competing financial interests or personal relationships that could have appeared to influence the work reported in this paper."
Availability of data, code and other materials	27	Report which of the following are publicly available and where they can be found: template data collection forms; data extracted from included studies; data used for all analyses; analytic code; any other materials used in the review.	Data Availability statement – "No data available"; extracted study data are presented in Table 2.

From: Page MJ, McKenzie JE, Bossuyt PM, Boutron I, Hoffmann TC, Mulrow CD, et al. The PRISMA 2020 statement: an updated guideline for reporting systematic reviews. BMJ 2021;372:n71. doi: 10.1136/bmj.n71. This work is licensed under CC BY 4.0. To view a copy of this license, visit https://creativecommons.org/licenses/by/4.0/
